# Profiling low-mRNA content cells in complex human tissues using BD Rhapsody single-cell analysis

**DOI:** 10.1016/j.xpro.2024.103475

**Published:** 2024-12-09

**Authors:** Alexandra Scheiber, Manuel Trebo, Annabella Pittl, Isabel Heidegger, Theresa Hautz, Rupert Oberhuber, Zlatko Trajanoski, Florian Augustin, Sieghart Sopper, Dominik Wolf, Andreas Pircher, Stefan Salcher

**Affiliations:** 1 Department of Internal Medicine V, Hematology and Oncology and Comprehensive Cancer Center Innsbruck (CCCI), Medical University of Innsbruck, 6020 Innsbruck, Austria; 2 Tyrolean Cancer Research Center Innsbruck, 6020 Innsbruck, Austria; 3 Department of Urology, Medical University of Innsbruck, 6020 Innsbruck, Austria; 4 Department of Visceral, Transplant and Thoracic Surgery, Center of Operative Medicine, organLife Laboratory and D. Swarovski Research Laboratory, Medical University of Innsbruck, 6020 Innsbruck, Austria; 5 Biocenter, Institute of Bioinformatics, Medical University of Innsbruck, 6020 Innsbruck, Austria; 6 Department of Visceral, Transplant and Thoracic Surgery, Medical University Innsbruck, 6020 Innsbruck, Austria

**Keywords:** Bioinformatics, Cancer, Sequencing, RNAseq

## Abstract

The successful recovery of immune cells, particularly those with low mRNA content, by single-cell RNA sequencing (scRNA-seq) remains a significant challenge. Tissue dissociation and selection of the appropriate scRNA-seq technology are crucial. Our protocol efficiently recovers low-mRNA content immune cells using the BD Rhapsody scRNA-seq platform. It includes optimized tissue dissociation for prostate, lung, and liver tissues, cell labeling with Sample Tag antibodies, microwell-based single-cell capture, cDNA synthesis, library preparation, and data pre-processing with basic quality control analysis.

For complete details on the use and execution of this protocol, please refer to Salcher et al.^1^

## Highlights

• Comprehensive protocol for single-cell profiling of low-mRNA content cells
• Optimized tissue dissociation for lung tumor, prostate tumor, and liver tissue
• Full single-cell RNA sequencing workflow, from sample preparation to data acquisition
• Guide for data processing and basic quality control

## Abstract (editor highlights)

Publisher’s note: Undertaking any experimental protocol requires adherence to local institutional guidelines for laboratory safety and ethics.

## Graphical abstract

**Figure:**
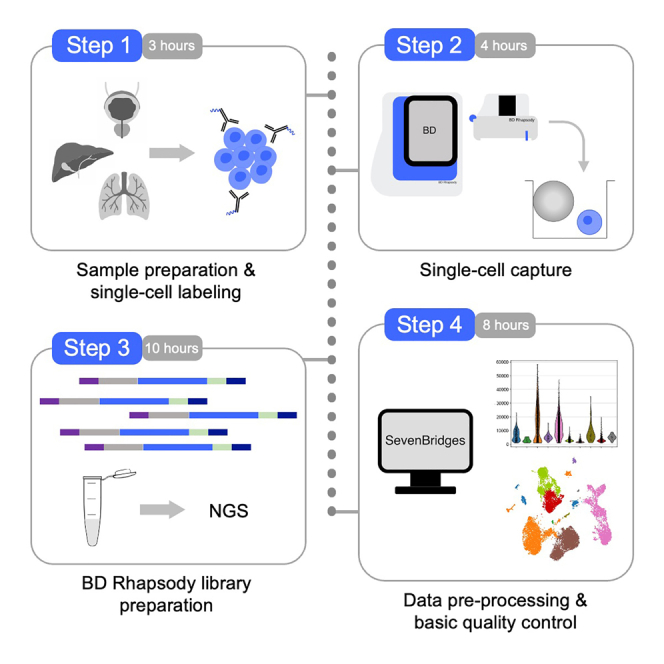

## Before you begin

**Figure 1:**
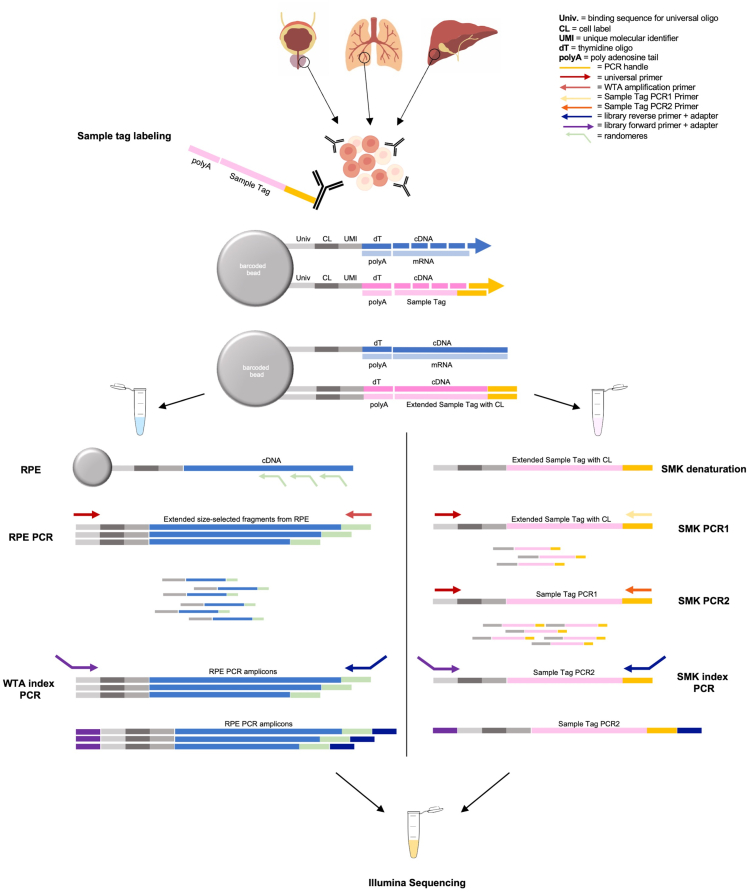
Schematic overview of wet lab workflow

The following protocol describes a whole workflow from preparation of single-cell suspensions to primary analysis of single-cell sequencing data (Figure 1). It is optimized to capture cells with low mRNA content (e.g., immune cells such as neutrophils or T cells) obtained from complex human tissues. We utilize the microwell-based BD Rhapsody single-cell sequencing analysis platform, for which significantly better recovery of low-mRNA content cells compared to droplet-based scRNA-seq has recently been demonstrated.^1^^,^^2^ The BD Rhapsody platform comprises several reagent kits, the cartridge kit, the BD Rhapsody Express instrument, and general consumables (see key resources table). We highly recommend using the BD Rhapsody scanner (part of this protocol) as it provides quality control checks during the process of sample preparation and cartridge loading. This enables more precise estimations of cell/bead capture rates, thus allowing optimized sequencing and downstream analysis. The labeling of single cells with universal antibody-oligonucleotides, so-called Sample Tags, is optional and enables the concurrent processing of multiple samples. Additionally, the BD Rhapsody platform also offers simultaneous analysis of mRNA and protein expression by using antigen-specific antibody-oligonucleotide conjugates, so-called AbSeqs (which is not part of this protocol, see^3^).

As this protocol extends over more than 8 h, stopping points have been incorporated to streamline time management. Overall, we estimate that the complete wet lab processing of the samples will require three full working days.

### Workspace requirements

Executing this protocol requires specific spatial arrangements. Tissue dissociation of human samples should be performed in a Biosafety Level 2 laboratory with a laminar airflow hood. Cartridge loading and preparation of PCR and other reaction mixes should be carried out in a pre-amplification workspace within a PCR cabinet. All PCRs and purifications must be conducted in a separate post-amplification room.

### Preparation of single-cell suspensions from primary tissues

Written informed consent must be obtained from all patients included in the study and the study has to be approved by the local ethical committee. Tissue samples carry a potential risk of being infectious, so special precautions must be taken, including wearing protective gloves, glasses, and a lab coat at all times. Upon tissue sampling, maintenance of tissue integrity is vital for securing cell quality and quantity of the obtained single-cell suspension. Keep the sample constantly on ice after extraction and pathological examination to reduce tissue damage. Acquiring freshly resected tissue samples is crucial when analyzing cells with very low mRNA content, such as neutrophils, as they are especially sensitive to handling procedures like freezing and thawing. You may adjust your dissociation protocol based on the specific tissue you are using. This protocol includes the dissociation of three different tissue types: prostate, lung and liver. The protocols for prostate and lung tissues have been tested on both normal/benign and tumor tissues. The described procedure enables the acquisition of high-quality single-cell transcriptomic data from cells with low mRNA content. For more detailed information on transcriptome analysis of these tissues, we recommend reading the original studies.^1^^,^^2^^,^^4^

### Preparation of reagents, buffers and solutions

Prepare all buffers and working solutions beforehand (see key resources table, materials and equipment). Thaw, vortex, and centrifuge all frozen reagents, then store them temporarily on ice except indicated otherwise. Enzymes should remain at −20°C until usage. Handle the BD Lysis Buffer and the BD Horizon Dri Tumor and Tissue Dissociation Reagent with great care.

### Institutional permissions

The collection of tissue samples used to conduct the described protocol was reviewed and approved by the Institutional Review Board at the Medical University of Innsbruck. The approval numbers for the studies are as follows: The prostate tissue study is approved under EK no. 1017/2018; 1072/2018. The lung tissue study is approved under AN214-0293 342/4.5. The liver tissue study is approved under EK no. 1175/2018. All patients participating in the studies provided written informed consent prior to any study-related procedures.

## Key resources table

**Table:** 

REAGENT or RESOURCE	SOURCE	IDENTIFIER
**Biological samples**		

Freshly resected benign prostate and prostate tumor tissue from patients with PCa	Salcher et al.^1^ Heidegger et al.^5^	https://doi.org/10.1016/j.heliyon.2024.e28358; https://doi.org/10.1186/s12943-022-01597-7
Freshly resected normal adjacent lung and lung tumor tissue from patients with NSCLC	Salcher et al.^2^	https://doi.org/10.1016/j.ccell.2022.10.008
Freshly resected liver tissue from healthy liver allografts	Hautz et al.^4^	https://doi.org/10.1038/s41467-023-37674-8

**Chemicals, peptides, and recombinant proteins**		

Bovine serum albumin	M&B Stricker/Pantech	Cat#: P30-193306
Collagenase type V	Sigma-Aldrich	Cat#: C9263 – 1G
Dispase II	Gibco	Cat#: 17105-041
DMEM high glucose	Sigma-Aldrich	Cat#: D6171
DPBS	Corning	Cat#: 21031CV
EDTA 1% w/o Ca, Mg (sterile filtered)	Pan-Biotech	Cat#: P10-026100
Ethyl alcohol absolute	VWR Chemicals	Cat#: 20821.321
HBSS	Cytiva	Cat#: SH30588.01
L-Glutamine–Penicillin–Streptomycin solution	Sigma-Aldrich	Cat#: G1146
Nuclease-free water (not DEPC-treated)	Invitrogen	Cat#: AM9939
DNase I	Sigma-Aldrich	Cat#: D4527

**Critical commercial assays**		

Agencourt AMPure XP magnetic beads	Beckman Coulter	Cat#: A63880
BD Horizon Dri Tumor and Tissue Dissociation Reagent	BD Biosciences	Cat#: 661563
BD Human Single-Cell Multiplexing Kit	BD Biosciences	Cat#: 633781
BD Pharm Lyse	BD Biosciences	Cat#: 555899
BD Rhapsody Cartridge Kit	BD Biosciences	Cat#: 633733
BD Rhapsody cDNA Kit	BD Biosciences	Cat#: 633773
BD Rhapsody Enhanced Cartridge Reagent Kit	BD Biosciences	Cat#: 664887
BD Rhapsody WTA Amplification Kit	BD Biosciences	Cat#: 633801
BD stain buffer (FBS)	BD Biosciences	Cat#: 554656
Calcein AM	Invitrogen	Cat#: C1430
Draq7	BD Pharmingen	Cat#: 564904
High sensitivity D1000 reagents	Agilent	Cat#: 5067–5585
High Sensitivity D1000 ScreenTape	Agilent	Cat#: 5067–5584
Qubit dsDNA HS Assay Kit	Invitrogen	Cat#: Q32854

**Deposited data**		

scRNA-seq raw data	Zenodo	Zenodo: 12164999; https://doi.org/10.5281/zenodo.12164999

**Software and algorithms**		

Seven Bridges BD Rhapsody Sequence Analysis Pipeline v1.2	SevenBridges	https://www.bdbiosciences.com/en-us/products/software/rhapsody-sequence-analysis-pipeline
python (3.12.3)	https://www.python.org/	https://www.python.org/
scanpy (1.10.1)	Wolf et al.^6^	https://scanpy.readthedocs.io/en/stable/
matplotlib (3.9.0)	Hunter^7^	https://pydata.org/project/matplotlib/
numpy (1.26.4)	Harris et al.^8^	https://pydata.org/project/numpy/
pandas (2.2.2)	McKinney^9^	https://pandas.pydata.org/
seaborn (0.13.2)	Waskom^10^	https://seaborn.pydata.org/

**Other**		

4200 TapeStation System	Agilent	Cat#: G2991BA
BD Rhapsody Express Instrument	BD Biosciences	Cat#: 633702
BD Rhapsody P1200M pipette	BD Biosciences	Cat#: 633704
BD Rhapsody P5000M pipette	BD Biosciences	Cat#: 633705
BD Rhapsody Scanner	BD Biosciences	Cat#: 633701
Cell strainer 100 μm	Falcon	Cat#: 352360
Cell strainer 70 μm	Falcon	Cat#: 352350
Centrifuge Rotina 420 R	Hettich Zentrifugen	Cat#: 4706
Disposable hemocytometer (Neubauer improved)	Incyto	Cat#: DHC-N01-5
DNA LoBind tubes 1.5 mL	Eppendorf	Cat#: 30108051
DNA LoBind tubes 5 mL	Eppendorf	Cat#: 30108310
Dynal MPC-1, magnetic separation rack 5 mL	Dynal A.S.	Cat#: 120.01
DynaMag-2, magnetic separation rack 1.5 mL	Invitrogen	Cat#: 12321D
Falcon 5 mL round-bottom polystyrene test tube	Corning	Cat#: 352054
Falcon tube with cell strainer Cap	Corning	Cat#: 352235
FastGene MagnaStand, magnetic separation rack 0.2 mL	Nippon Genetics Europe	Cat#: FG-SSMAG2
FlexCycler thermocycler	Analytik Jena	N/A
GentleMACS C tube	Miltenyi Biotec	Cat#: 130-093-237
GentleMACS Octo Dissociator with heaters	Miltenyi Biotec	Cat#: 130-096-427
Low-retention filter pipette tips 10 μL	Biozym	Cat#: VT0200
Low-retention filter pipette tips 100 μL	Biozym	Cat#: VT0230
Low-retention filter pipette tips 1250 μL	Biozym	Cat#: VT0270
Low-retention filter pipette tips 200 μL	Biozym	Cat#: VT0240
Minicentrifuge for 0.2 mL PCR tube strip	Major supplier	N/A
Minicentrifuge for 1.5 mL tubes	Major supplier	N/A
PCR tube strip 0.2 mL	Major supplier	N/A
Petri dish	Falcon	Cat#: 353003
Qubit Assay tubes	Invitrogen	Cat#: Q32856
Qubit fluorometer	Invitrogen	Cat#: Q33238
Serological pipettes 10 mL	Major supplier	N/A
Serological pipettes 25 mL	Major supplier	N/A
Serological pipettes 5 mL	Major supplier	N/A
Serological pipettes 50 mL	Major supplier	N/A
SmartBlock 1.5 mL	Eppendorf	Cat#: 5360000038
Sterile forceps	Major supplier	N/A
Sterile scissors	Major supplier	N/A
Thermocycler	Major supplier	N/A
ThermoMixer C	Eppendorf	Cat#: 5382000015
Tubes 50 mL	Sarstedt	Cat#: 62.547.254

## Materials and equipment

### Preparation of buffers and solutions

**Table:** Prostate dissociation mix

Reagent	Stock concentration	Amount
DNase I	75 U/mL	20 μL
Collagenase Type V	2%	1 mL
Dispase II	2.5 U/mL	1 mL
DMEM high glucose + 1% L-glutamine-penicillin-streptomycin (100 U/mL)	N/A	8 mL

Sterile-filtered, do not store, prepare fresh.

**Table:** Working buffer

Reagent	Stock concentration	Amount
BSA in DPBS	10%	10.00 mL
EDTA in DPBS	1%	5.88 mL
DPBS	N/A	84.12 mL

Sterile-filtered, store at 4°C, up to 1 week.

• Erythrocyte lysis buffer: prepare 1x solution by diluting 10x BD Pharm Lyse lysing buffer with ddH_2_O (do not store, prepare fresh).
• Calcein AM: dissolve the Calcein AM with 503 μL of DMSO (store at −20°C, up to 6 months).
• 80% ethyl alcohol: add 2 mL of nuclease-free water to 8 mL of absolute ethyl alcohol (store at RT, up to 24 h).

### Equipment setup

• Use Agilent DNA High Sensitivity Kit or Agilent High Sensitivity D5000 ScreenTape and Reagents instead of Agilent High Sensitivity D1000 ScreenTape and Reagents.
• Use Agilent 2100 Bioanalyzer instead of Agilent 4200 TapeStation System.
• Eppendorf ThermoMixer C with 1.5 mL SmartBlock (Alternative: any programmable thermomixer): Create and save the following protocol before you start. Ensure to use “time control” as time mode.
  ○ step 1: 25°C for 10 min at 1,200 rpm.
  ○ step 2: 37°C for 15 min at 1,200 rpm.
  ○ step 3: 45°C for 10 min at 1,200 rpm.
  ○ step 4: 55°C for 10 min at 1,200 rpm.
• Thermocycler: Create and save the PCR programs before you start. See “step-by-step method details” section for PCR cycling conditions.
• Software requirements: Anaconda environment with python 3.12.3, scanpy 1.10.1, matplotlib 3.9.0, seaborn 0.13.2, numpy 1.26.4, pandas 2.2.2 (Alternatives: R/R Studio with Seurat).^11^

## Step-by-step method details

The first three sections provide detailed preparation protocols specific to each tissue type: prostate, lung, and liver. Select the protocol that best suits your specific tissue. From the section “cell counting” onward, the protocol is consistent for all three tissue types.

### Preparation of single-cell suspensions of prostate tissue

**Timing: 1 h 15 min**

In this section we describe the dissociation of prostate tissue to get single cells. Benign and tumor prostate tissue is obtained from radical prostatectomy samples. From a central slice benign and tumor areas are punched to get 0.8 cm biopsies.

1. Prepare the prostate dissociation mix and working buffer according to the recipe in the “materials and equipment” section.

***Note:*** Use 10 mL of prostate dissociation mix per each prostate tissue sample.

2. Dissect the tissue with mechanical and enzymatic dissociation.
  a. Place the tissue into a petri dish and rinse with 5 mL cold DPBS once.
  b. Drain the biopsies and transfer into a new petri dish.
  c. Add 5 mL of the freshly prepared dissociation mix.
  d. Cut the tissue into small pieces (< 1 mm) with a sterile surgical scissor or a scalpel blade.
  e. Pipette the solution up and down and transfer it to a 50 mL Falcon tube.
  f. Wash petri dish with 5 mL of dissociation solution and add the solution to the Falcon tube.
  g. Place the tube into a preheated water bath at 37°C for 45 min.
  h. Gently shake the tube every 5 min.
  i. Immediately stop digestion by adding 5 mL working buffer to the digestion tube.
3. Strain and wash cells.
  a. Place a 100 μm cell strainer on top of a 50 mL tube and filter cells through.
  b. Rinse the original 50 mL tube with 5 mL working buffer and transfer onto the filter.
  c. Repeat step 3b.
  d. Centrifuge the cell suspension at 4°C for 5 min at 300 × *g*.
  e. Resuspend the pellet in 1 mL BD Sample Buffer.

***Optional:*** To enrich specific cell populations, perform fluorescent-activated cell sort after dissociation. If Sample Tag labeling is performed, resuspend the pellet in 1 mL BD Stain Buffer.

### Preparation of single-cell suspensions of lung tissue

**Timing: 2 h**

This section describes the dissociation of lung tissue to get single cells. Lung tissue is obtained from lobectomy samples. Normal and tumor tissue slices of 0.3–1 g are processed.

4. Prepare the working buffer and the BD Pharm Lyse lysing buffer according to the “materials and equipment” section. Dissolve the BD Horizon Dri Tumor and Tissue Dissociation Reagent (TTDR).
  a. Dissolve the lyophilized reagent with 5 mL of warm HBSS.
  b. Keep in an incubator at 37°C for a maximum of 30 min.
    **CRITICAL:** Wear protective glasses, gloves and a lab coat when handling the tumor dissociation reagent.
    **CRITICAL:** Do not exceed incubation time of BD TTDR at 37°C.
5. Dissect the tissue with mechanical and enzymatical dissociation.
  ***Note:*** Use 1 tube of BD TTDR for 0.1–1 g of tumor tissue and 1 tube for 0.1–1.5 g of normal lung tissue.
  a. Add 5 mL of HBSS to a gentleMACS C tube.
  b. Weigh the drained tissue using a petri dish.
  c. Transfer the tissue into the tube using forceps.
  d. Cut the tissue into small pieces with surgical scissors.
  e. Combine 5 mL of TTDR with 5 mL HBSS in the gentleMACS C tube (Figure 2).

**Figure 2:**
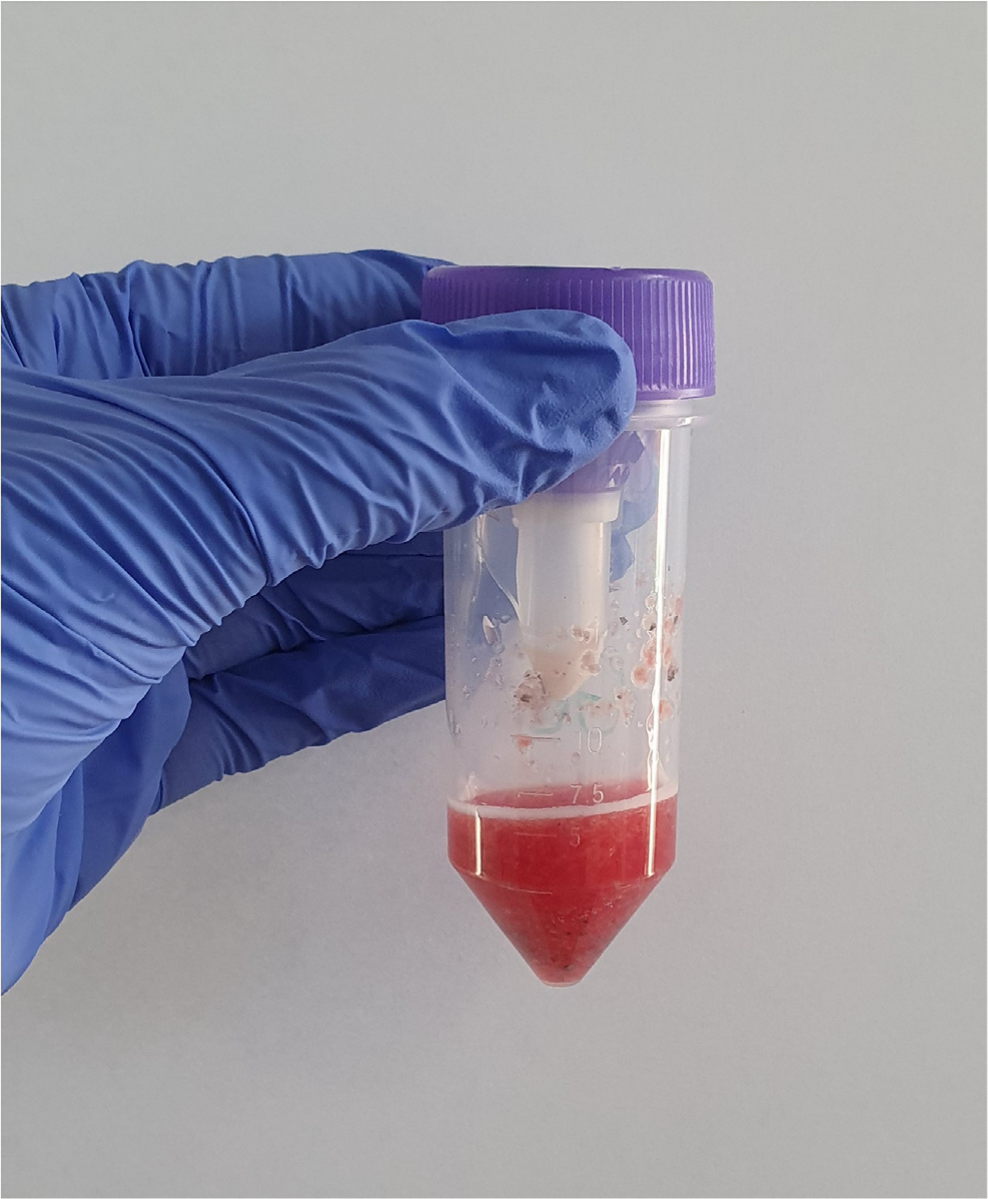
GentleMACS tube with dissociated tissue
  f. Start the gentleMACS program h_tumor_01.
  g. Remove gentleMACS C tube and incubate at 37°C for 15 min at 150 rpm.
  h. Repeat step 5f.-g. once more using program h_tumor_02.
  i. Finish dissociation with program h_tumor_03.
6. Strain and wash cells.
  a. Place a 100 μm cell strainer on top of a 50 mL tube and filter the cell suspension through.
    ***Note:*** Use the plunger of a 5 mL syringe to gently press the remaining tissue parts through the filter (Figure 3).

**Figure 3:**
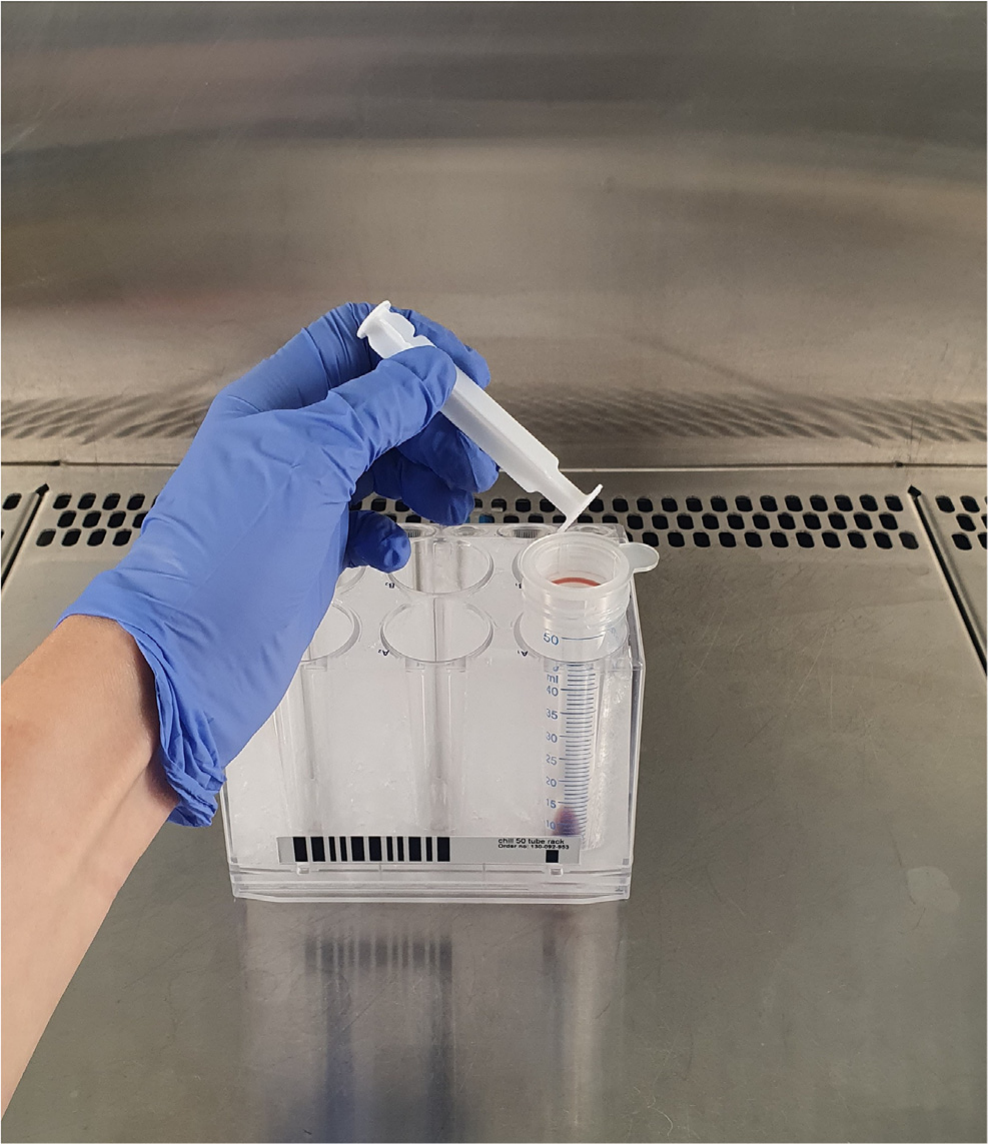
Cell straining with plunger
  b. Rinse the gentleMACS C tube with 5 mL working buffer and transfer onto the filter.
  c. Repeat this step one more time.
  d. Fill up the 50 mL tube with working buffer to 45 mL.
  e. Centrifuge the cell suspension at 10°C for 10 min at 300 × *g*.
  f. Discard the supernatant and resuspend the cell pellet with 2–3 mL erythrocyte lysing buffer.
  g. Incubate in the dark at 20°C for 10 min.
  h. Fill up the tube with 20–40 mL cold HBSS and centrifuge cell suspension at 4°C for 8 min at 300 × *g*.
  i. Aspirate the supernatant and add 1 mL of BD Sample Buffer.
  j. Repeat filter step using a 70 μm cell strainer and a fresh 50 mL tube.
  k. Transfer the cell suspension onto the filter.
  l. Add 1 mL BD Sample Buffer to the original 50 mL tube and transfer onto the filter.
  m. Repeat two more times to get a final volume of approximately 4 mL.
    ***Optional:*** If Sample Tag labeling is performed, resuspend the pellet in 1 mL BD Stain Buffer.

### Preparation of single-cell suspensions of liver tissue

**Timing: 2 h**

The following section describes the dissociation of liver tissue to get single cells. The liver tissue is obtained from healthy liver allografts. Liver biopsies of 0.1–0.5 g are processed.

7. Prepare the working buffer and the BD Pharm Lyse lysing buffer according to the “materials and equipment” section. Dissolve the BD Horizon Dri Tumor and Tissue Dissociation Reagent (TTDR) and prepare pipette tips.
  a. Dissolve the lyophilized reagent with 5 mL of warm HBSS.
  b. Keep in an incubator at 37°C for a maximum of 30 min.
  c. Cut off 5 mm from the narrow ends of sterile 1000 μL pipette tips with some scissors.

**CRITICAL:** Wear protective glasses, gloves and a lab coat when handling the tumor dissociation reagent.

**CRITICAL:** Do not exceed incubation time of BD TTDR reagent at 37°C.

8. Dissect the tissue with mechanical and enzymatic dissociation.
  ***Note:*** Use 1 tube of BD TTDR for 0.1–0.5 g of tissue.
  a. Weigh the drained tissue using a petri dish.
  b. Add 2 mL of cold HBSS to the petri dish with the liver tissue.
  c. Cut the tissue into small pieces (< 1 mm) with a sterile surgical scissor or a scalpel blade.
  d. Transfer the tissue into a 15 mL Falcon tube using the cut pipette tips from step 7c.
  e. Add 2 mL of warm TTDR into the tube.
  f. Incubate the tube at 37°C for 20 min at 150 rpm.
  g. After finishing dissociation, place the tube on ice to stop digestion.
  h. Add 0.8 mL cold HBSS to the tube.
  i. Use cut pipette tips to extract cells from the tissue pieces by rigorously pipetting up and down.
  j. Perform step 8i. until the solution turns cloudy/brown.
  k. Let the tissue pieces sink to the bottom of the tube and transfer supernatant to a new 15 mL tube on ice.
  l. Add 1.6 mL cold HBSS to the tissue pieces and repeat step 8i.-k. until no more cells can be extracted.
    ***Note:*** Cell extraction can be visually evaluated by the whitening of tissue pieces within the solution during step 8l.
9. Strain and wash cells.
  a. Centrifuge the cell suspension at 4°C for 8 min at 300 × *g*.
  b. Discard the supernatant and resuspend the cell pellet with 2–3 mL erythrocyte lysing buffer.
  c. Incubate in the dark at 20°C for 10 min.
  d. Fill up the tube with 15 mL cold HBSS.
  e. Centrifuge the cell suspension at 4°C for 5 min at 300 × *g*.
  f. Aspirate the supernatant and add 1 mL of BD Sample Buffer to resuspend cell pellet.
  g. Place a 70 μm cell strainer on top of a 50 mL tube and filter the cells through.
  h. Add 1 mL BD Sample Buffer to the original tube and transfer onto the filter to get a final volume of approximately 2 mL.

***Optional:*** If Sample Tag labeling is performed, resuspend the pellet in 1 mL BD Stain Buffer.

### Cell counting

**Timing: 15 min**

In this section we describe how to count cells obtained from different tissues with the BD Rhapsody scanner.

10. Determine the number of viable cells.
  a. Prepare a 1.5 mL tube and dilute the cells 1:10 in 100 μL BD Sample Buffer.
  b. Add 0.5 μL of Draq7 and 0.5 μL of Calcein AM to the diluted cell suspension.
  c. Incubate sample on the thermomixer at 37°C for 5 min at 400 rpm in the dark.
  d. Pipet-mix cell suspension and use 10 μL to load the INCYTO disposable hemocytometer.
  e. Start the BD Rhapsody scanner and open the BD Rhapsody software. Tap “Scan” to continue.
  f. Insert the hemocytometer with corresponding adapter into the BD Rhapsody Scanner and tap “Continue”.
  g. Define the experiment, sample and username and select the hemocytometer protocol.
  h. After measurement, the instrument’s analysis software provides the cell concentration (cells per μL) and viability percentage.
  i. Use the total volume of your cell suspension to calculate the total amount of viable cells obtained. See troubleshooting 1.

***Note:*** If the cell number is too high for reliable cell counting with the scanner (cells per μL > 1000) add 100 μL of Sample Buffer or Stain Buffer to the stained cell suspension and immediately repeat cell count step. If the cell number is too low (cells per μL < 100) prepare a new cell suspension using a lower dilution and repeat cell counting step.

### Single-cell labeling with Sample Tags (optional)

**Timing: 45 min**

Single-cell labeling using Sample Tags allows for the combination of up to 12 individual samples onto one BD Rhapsody Cartridge. Each sample is labeled with a unique Sample Tag, enabling them to be mixed and sequenced together. This antibody-oligo approach utilizes a barcoding system, facilitating high-throughput library preparation. Sample demultiplexing is performed during data analysis.

***Note:*** Use 300,000–500,000 viable cells per each sample. If the cell count is lower, follow the recommendations in the section below. Use the human BD Single-Cell Multiplexing Kit (SMK) and BD Enhanced Cartridge Reagent Kit. This step is optional. If no Sample Tag labeling is performed, continue with the next step “preparation, loading and capture of single cells with the BD Rhapsody single-cell analysis system” immediately after cell counting.

11. Label cells with the BD single-cell multiplexing kit.
  a. Adjust the cell number to 300,000–500,000 of living cells and transfer the cell suspension into a new 5 mL polystyrene tube.
    ***Note:*** If the volume is below 180 μL bring volume to 180 μL and continue with step 11e. Otherwise continue with step 11b.
  b. Centrifuge at 20°C for 5 min at 400 × *g*.
  c. Invert the tubes to decant and remove any residual liquid from the rim with a wiper.
    **CRITICAL:** To not invert the tubes between the decantation and liquid removal, as it might loosen the pellet.
  d. Add 180 μL of BD Stain Buffer and resuspend each pellet.
  e. Transfer the whole amount of one Sample Tag tube (20–30 μL) into the corresponding 5 mL tube.
  f. Incubate at 20°C for 20 min.
    ***Note:*** During incubation start with the preparation of the BD Rhapsody Cartridge (see step 14 under “preparation, loading and capture of single cells with the BD Rhapsody single-cell analysis system”).
12. Wash the Sample Tag-labeled cells.
  a. Add 2 mL of BD Stain Buffer to the labeled cell suspension.
  b. Centrifuge at 4°C for 5 min at 400 × *g*.
  c. Decant the tubes and resuspend the pellet in 2 mL BD Stain Buffer.
  d. Repeat steps 12b.-c. two more times for a total of three washes.
  e. After last step of centrifugation add 620 μL of BD Sample Buffer and put labeled cells on ice.
    ***Note:*** We recommend three rounds of washing. Each round results in substantial cell loss. Therefore, for samples with limited cell numbers the number of washing steps can be reduced. However, this might result in unspecific staining and an increased proportion of undetermined cells during sample demultiplexing due to insufficient Sample Tag information.
    **CRITICAL:** Never vortex Sample Tag-labeled cells.
  f. Count the cells using the hemocytometer and the Rhapsody Scanner.
    i. Add 3.1 μL of Draq7 and 3.1 μL of Calcein AM to the cell suspension of step 12e.
    ii. Incubate sample on the thermomixer at 37°C for 5 min at 400 rpm in the dark.
    iii. Load 10 μL into the INCYTO disposable hemocytometer and measure cell concentration with the BD Rhapsody scanner as described in step 10 of “cell counting”.

### Preparation, loading and capture of single cells with the BD Rhapsody single-cell analysis system

**Timing: 2 h 30 min**

In this section the loading and capture of single cells with BD Rhapsody Enhanced Capture Beads is described. Up to 40,000 cells can be loaded in one BD Rhapsody cartridge. After lysis of the cells, the mRNA is bound to the beads and retrieved from the cartridge.

13. Prepare the single-cell suspension and reagents.
  a. Put the BD Stain Buffer, Sample Buffer, Bead Wash Buffer, Lysis Buffer, DTT and Enhanced Beads on ice and the Cartridge Wash Buffer 1 and 2 to RT.
  b. If the concentration is above 1,000 cells per μL adjust the volume of the sample by adding another 100–600 μL of BD Sample Buffer.
  c. Use the “Prepare” function of the analysis software and tap to open.
  d. Define your total number of cells captured and prepare the cell suspension according to the volumes given on the display of the scanner.
    ***Note:*** Volumes provided by the sample calculator might result in lower capture rates than expected, as it does not account for cell viability. If using Sample Tag-labeled cells adjust the relative amounts of the sample with lower viability (Figure 4).

**Figure 4:**
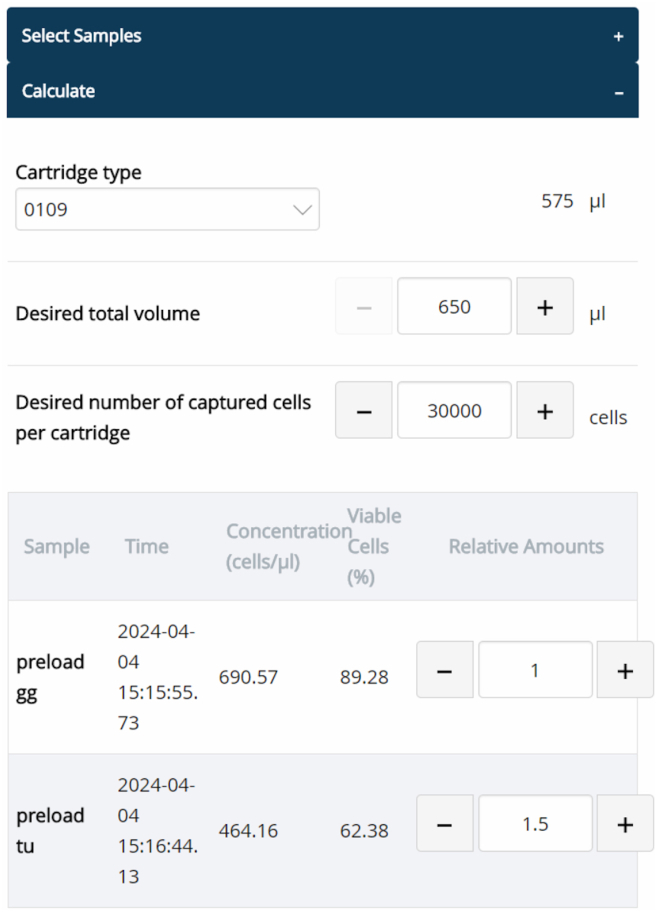
Sample calculator of BD Rhapsody Scanner for cartridge loading Ratio of samples adjusted to viability.
  e. Filter the total volume of 650 μL through a Falcon tube with a cell strainer cap. Keep dark and on ice until loading onto the cartridge.
14. Priming of the BD Rhapsody Cartridge.
  a. Place the cartridge on the scanner.
  b. Add a waste container and a 5 mL LoBind Tube to the Express instrument drawer.
  c. Set the electronic BD Rhapsody P1200M pipette to the “Prime/Treat” mode.
  d. Place the side slider of the Express instrument to “0” and the front slider to the “Waste” position.
  e. Load the cartridge with 700 μL of 100% ethanol followed by 700 μL of air and 700 μL of BD Cartridge Wash Buffer 1.
  f. Incubate the BD Rhapsody Cartridge for 1 min and remove the Wash Buffer 1 with 700 μL of air.
  g. Continue with another 700 μL of BD Cartridge Wash Buffer 1 and incubate 10 min.
  h. Remove the Wash Buffer 1 from the cartridge with 700 μL of air.
  i. Load 700 μL BD Rhapsody Cartridge Wash Buffer 2.

**CRITICAL:** The pipette tip must be sealed airtight to ensure optimal volume and flow velocity.

***Note:*** Use the primed cartridge within 4 h. Store at 20°C and on the Express instrument.

**Figure 5:**
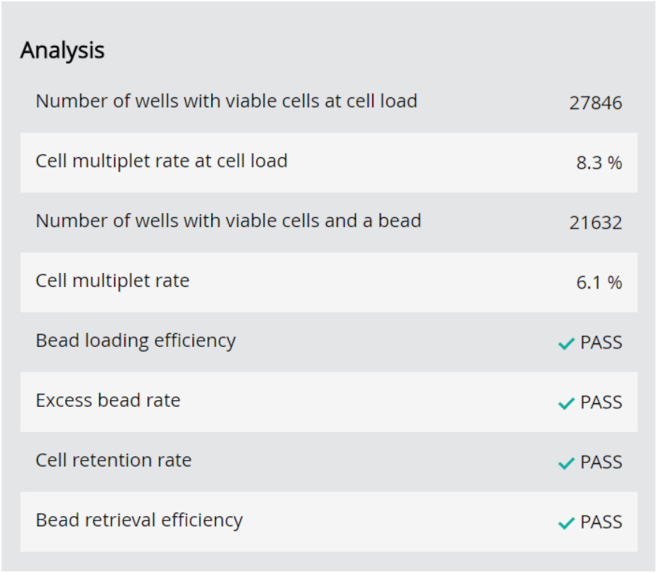
BD Cartridge image metrics

15. Loading of the cells onto the BD Rhapsody Cartridge.
  a. Remove the Cartridge Wash Buffer 2 by loading 700 μL of air.
  b. Change the pipette mode of the P1200M to “Cell Load” and resuspend the cell suspension with a conventional pipette by carefully pipetting up and down.
    **CRITICAL:** During the cell loading step the button of the P1200M pipette needs to be pressed three times. Press once to aspirate 40 μL of air and again to aspirate 575 μL of cell suspension. Then press again to load 615 μL into the cartridge.
  c. Load 575 μL of the cell suspension onto the BD Rhapsody Cartridge.
  d. Start a timer and place the Rhapsody Cartridge on the scanner.
  e. Incubate for 20 min and enter time delay if necessary.
    **CRITICAL:** We recommend extending the 15 min sedimentation time (described in the original BD Rhapsody protocol) by 5 min to effectively capture small cells, such as immune cells/neutrophils, in the microwells.
  f. During incubation prepare the Enhanced Capture Beads.
    i. Place the Beads on a 1.5 mL magnetic rack and let them settle for 1 min.
    ii. Remove the storage buffer while tube is still on the magnet.
    iii. Remove tube from the magnet and add 750 μL of cold Sample Buffer to the beads. Pipet up and down, and place tube on ice until used.
      **CRITICAL:** Never vortex the beads.
  g. During incubation prepare the Lysis Buffer.
    i. Add 75 μL of 1 M DTT to one bottle of Lysis Buffer.
    ii. Vortex 10 s and place on ice.
  h. Press “Start Cell Load Scan”.
  i. After scanning, place the BD Rhapsody Cartridge back on Express instrument.
16. Loading of the BD Rhapsody Enhanced Cell Capture Beads.
  a. Change the pipette mode of the P1200M to “Prime/Treat” and load 700 μL of air.
  b. Resuspend the BD Rhapsody Enhanced Capture Beads with a conventional pipette by carefully pipetting up and down.
  c. Change the pipette mode of the P1200M to “Bead Load” and aspirate 630 μL of the bead suspension.
  d. Load 630 μL of the bead suspension onto the BD Rhapsody Cartridge.
  e. Set timer to 3 min and place the BD Rhapsody Cartridge on the scanner.
  f. Start “Bead Load Scan” after incubation time is over.
  g. After scanning, place the BD Rhapsody Cartridge back on the Express instrument.
17. Washing of the BD Rhapsody Enhanced Cell Capture Beads.
  a. Change the pipette mode of the P1200M to “Wash”.
    **CRITICAL:** During the washing step the button of the P1200M pipette needs to be pressed three times. Press once to aspirate 720 μL of the Bead Wash Buffer. Press again to load 700 μL into the cartridge and again to dispense 20 μL in the waste.
  b. Remove the Bead Suspension by loading 700 μL of air.
  c. Load 700 μL of cold Sample Buffer.
  d. Repeat step 17b.-c. one more time.
  e. Place the BD Rhapsody Cartridge on the scanner and start “Bead Wash Scan”.
  f. After scanning, place the BD Rhapsody Cartridge back on Express instrument.
18. Lysing the cells and retrieving the BD Rhapsody Enhanced Cell Capture Beads.
  a. Change the pipette mode of the P1200M to “Lysis”.
  b. Place the side slider of the Express instrument to “Lysis”. The front slider remains at the “Waste” position.
  c. Load the cartridge with 550 μL of Lysis Buffer of step 15g.
  d. Incubate at 20°C for 2 min and place the front slider of the Express instrument to “Beads”.
    ***Note:*** Wear protective glasses, gloves and lab coat while handling with the BD Lysis Buffer.
    **CRITICAL:** Do not exceed the incubation time and avoid bubbles.
  e. After incubation, move the side slider of the Express instrument to “Retrieval”.
  f. Set timer to 30 s and immediately start with aspirating 5,000 μL prepared BD Lysis Buffer with the P5000M pipette.
  g. After incubation is over, immediately move side slider to “0” and load the BD Rhapsody Cartridge with 4,950 μL prepared BD Lysis Buffer.
  h. Remove the 5 mL LoBind Tube from the drawer and place on a 5 mL tube magnet.
  i. Incubate for 1 min and remove 4 mL of supernatant.
  j. During incubation place the BD Rhapsody Cartridge on the scanner and start the “Retrieval Scan”.
  k. After incubation wash the Cell Capture Beads.
    i. Remove the 5 mL tube from the magnet and transfer the remaining lysis buffer and the beads to a new 1.5 mL tube.
    ii. Place the tube on a magnet for 2 min and carefully withdraw the supernatant.
    iii. Remove the 1.5 mL tube from the magnet and resuspend the beads with 1 mL cold Bead Wash Buffer.
    iv. Repeat steps ii. and iii. one more time. Place the tube on ice for max. 30 min. Analyze the BD Cartridge image metrics (Figure 5).
      **CRITICAL:** Avoid bubbles in all steps. If beads remain in the 5 mL tube after transfer, add 0.5 mL prepared BD Lysis Buffer, resuspend and transfer beads to the 1.5 mL.

### Reverse transcription and exonuclease I treatment

**Timing: 1 h 20 min**

After lysis the mRNA is bound to the 3′ end of the barcoded oligo of the bead via the poly-A tail. The cDNA is synthesized on the bead. During reverse transcription the barcode information is also added to the Sample Tags. The Cell Capture Beads are treated with Exonuclease I to remove residual primers and oligos from the reverse transcription.

19. Pre-heat two thermomixers, one at 37°C and one at 80°C. Ensure all reagents from the BD Rhapsody cDNA Kit are fully thawed, vortexed and centrifuged. Place all tubes on ice. The Reverse Transcriptase and the Exonuclease I should be kept on −20°C until usage.
20. Prepare the cDNA mix (Table 1) by combing all reagents into one 1.5 mL LoBind tube. Vortex mix, briefly centrifuge and place on ice.

**Table 1:** cDNA mix

Component	Amount [μl] for 1 sample incl. 20% overage
Nuclease-free water	117.6
RT Buffer	48.0
dNTP	24.0
Bead RT/PCR Enhancer	14.4
RT 0.1 M DTT	12.0
RNase Inhibitor	12.0
Reverse Transcriptase	12.0
21. Place the 1.5 mL tube from step 18k. on the magnet for 2 min and withdraw the supernatant.
22. Remove the tube from the magnet and resuspend the beads in 200 μL cDNA mix. Transfer to a new 1.5 mL LoBind Tube.
23. Incubate at 37°C for 20 min at 1,200 rpm.

**CRITICAL:** Shaking is essential at this step.

24. Prepare the Exonuclease I mix (Table 2) by combing all reagents into one 1.5 mL LoBind tube. Vortex mix, briefly centrifuge and place on ice.

**Table 2:** Exonuclease I mix

Component	Amount [μl] for 1 sample incl. 20% overage
Nuclease-free water	204.0
10x Exonuclease I Buffer	24.0
Exonuclease I	12.0
25. After incubation place the 1.5 mL tube onto the magnet and withdraw the supernatant.
26. Remove the tube from the magnet and resuspend the beads in 200 μL Exonuclease I mix.
27. Incubate at 37°C for 30 min at 1,200 rpm.

**CRITICAL:** Shaking is essential at this step.

28. Transfer tube to the other pre-heated thermomixer. Incubate at 80°C for 20 min with no shaking.
29. Incubate on ice for 1 min.
30. After incubation place the 1.5 mL tube on the magnet for 1 min and withdraw the supernatant.
31. Remove the tube from the magnet and resuspend the beads in 200 μL Bead Resuspension Buffer.

**Pause point:** Store the Exonuclease I-treated Cell Capture Beads at 4°C and start with the library preparation within 48 h.

### Random priming and extension, Sample Tag PCR1 and Sample Tag PCR2

**Timing: 6 h**

In this section we describe the Random Priming and Extension (RPE), Sample Tag PCR1 and PCR2. The Sample Tags are denaturated from the beads and amplified twice with Sample Tag-specific primers. During denaturation also the mRNA is removed from the cDNA-mRNA-duplex. The whole transcriptome amplification (WTA) sequencing library is generated from the remaining single-stranded cDNA using random priming and extension.

***Note:*** If no Sample Tag labeling has been performed skip all optional steps. Prepare all master mixes in the pre-PCR amplification workspace. Run PCR and purify the PCR products in the post-PCR workspace.

32. Pre-heat three thermomixers at 25°C, 37°C and 95°C and a programmable thermomixer (see “materials and equipment” section). Ensure all the reagents from the BD Rhapsody WTA Kit are fully thawed, vortexed and centrifuged. Place all tubes on ice. The WTA Extension Enzyme should be kept on −20°C until usage.
  a. Nuclease-free water.
  b. WTA Extension Buffer.
  c. WTA Extension Primers.
  d. 10 mM dNTP.
  e. Bead RT/PCR Enhancer.
  f. PCR Master Mix.
  g. Universal Oligo.
  h. Sample Tag PCR1 Primer (optional).
  i. Sample Tag PCR2 Primer (optional).
  j. WTA Amplification Primer.
  k. Elution Buffer.
  l. Bead Resuspension Buffer.
  m. WTA Extension Enzyme.
33. Prepare the Random Primer mix (Table 3) by combing all reagents into one 1.5 mL LoBind tube. Pipet-mix, briefly centrifuge and keep on RT.

**Table 3:** Random Primer mix

Component	Amount [μL] for 1 sample incl. 20% overage
Nuclease-free water	160.8
WTA Extension Buffer	24.0
WTA Extension Primers	24.0
34. Pipet-mix the Exonuclease I-treated Cell Capture Beads from step 31.

***Optional:*** Sub-sample Exonuclease I-treated Cell Capture Beads by calculating the volume of the desired cell number. Adjust total volume to 200 μL with Bead Resuspension Buffer. Store remaining beads at 4°C.

35. (Optional) Place on the magnet for a maximum of 2 min.
36. (Optional) Withdraw supernatant and remove the tube from the magnet.
37. (Optional) Resuspend with 75 μL Elution Buffer and pipet-mix 10 times.
38. Incubate at 95°C for 5 min.
39. Centrifuge the tube and place on the magnet for max. 2 min.
40. (Optional) Transfer supernatant in a fresh 1.5 LoBind tube (“Sample Tag”) and place on ice.
41. Wash the beads through removing the tube from the magnet and resuspending the beads with 200 μL Elution Buffer.
42. (Optional) Pipet-mix 10 times, centrifuge and place the tube back on the magnet for max. 2 min.
43. Withdraw the supernatant.
44. Remove the tube with the beads from the magnet and add 87 μL of the Random Primer mix into it. Thoroughly mix by pipetting up and down 10 times to resuspend the beads.
45. Start incubation on the thermomixers.
  a. First incubation: 95°C for 5 min, without shaking.
  b. Second incubation: 37°C for 5 min at 1,200 rpm.
  c. Third incubation: 25°C for 5 min at 1,200 rpm.
    **CRITICAL:** Shaking is essential at these steps.
  d. Keep tube at 20°C until proceeding with next step.
46. Prepare the Primer Extension mix (Table 4) by combing all reagents into one 1.5 mL LoBind tube. Pipet-mix, briefly centrifuge and place on ice.

**Table 4:** Primer Extension mix

Component	Amount [μl] for 1 sample incl. 20% overage
Bead RT/PCR Enhancer	14.4
10 mM dNTP	9.6
WTA Extension Enzyme	7.2
47. After incubation, centrifuge the tube and add 13 μL of Primer Extension mix.
48. Incubate on the thermomixer.
  a. First incubation: 25°C for 10 min at 1,200 rpm.
  b. Second incubation: 37°C for 15 min at 1,200 rpm.
  c. Third incubation: 45°C for 10 min at 1,200 rpm.
  d. Forth incubation: 55°C for 10 min at 1,200 rpm.

**CRITICAL:** Shaking is essential at these steps.

49. (Optional) During incubation start the Sample Tag PCR1 run.
  a. Prepare the Sample Tag PCR1 mix (Table 5) in pre-amplification workspace by combing all reagents in one 1.5 mL LoBind tube. Vortex-mix, briefly centrifuge and place on ice.

**Table 5:** Sample Tag PCR1 mix

Component	Amount [μl] for 1 sample with no overage
PCR Master Mix	100.0
Universal Oligo	20.0
Nuclease-free water	12.0
Sample Tag PCR1 Primer	1.0
  b. Add 67 μL of the “Sample Tag” (step 40) to the Sample Tag PCR1 mix tube and pipet-mix 10 times.
    **CRITICAL:** Vortexing of the Sample Tag/Sample Tag PCR1 mix tube has a negative effect on the outcome of the Sample Tag PCR.
  c. Distribute the Sample Tag reaction mix to four PCR tubes à 50 μL.
  d. Bring PCR strip to post-PCR amplification workspace, centrifuge and start the thermal cycler according to following program (Table 6).
    ***Note:*** The number of PCR cycles depend on the number of cells retrieved from the cartridge. We recommend 10 cycles for >20,000 cells, 11 cycles for >10,000 and 12 cycles for >5,000 cells.
    **Pause point:** Run PCR overnight at 4°C.

**Table 6:** PCR cycling conditions for Sample Tag PCR 1

Step	Temperature	Time	Cycles
Hot Start	95°C	3 min	1
Denaturation	95°C	30 s	10–15x
Annealing	60°C	3 min	
Extension	72°C	1 min	
Final extension	72°C	5 min	1
Hold	4°C	N/A	N/A
50. After incubation continue with Random Priming and Extension and place the tube on the magnet for a maximum of 2 min and discard supernatant.
51. Take the tube off the magnet and add 205 μL of Elution Buffer.
52. Pipet-mix until beads are resuspended.
53. Incubate the tube on a thermomixer at 95°C for 5 min without shaking.
54. After incubation resuspend beads with a thermomixer at any temperature for 10 s at 1,200 rpm.
55. Place the tube on the magnet and label a new 1.5 mL LoBind tube with “RPE product”.
56. Pipette 200 μL of supernatant into the newly labeled tube and place at ice.
57. Repeat “random priming and extension, Sample Tag PCR1 and Sample Tag PCR2” steps 44–56 (omitting step 49).
58. Add 200 μL of Bead Resuspension Buffer to the leftover Exonuclease I-treated Cell Capture Beads and store at 4°C.
59. Purify the RPE product (400 μL) with a single-sided AMPure cleanup.
  ***Note:*** Work in post-amplification workspace. AMPure beads should be brought to 20°C and vortexed at full speed for 1 min before use.
  **CRITICAL:** Stick to the sample volumes and adjust with Elution Buffer if necessary. Do not exceed the incubation times.
  a. Prepare 10 mL of fresh 80% ethyl alcohol (see “materials and equipment”).
  b. Add 720 μL of AMPure beads to the RPE product and pipet-mix 10 times.
  c. Incubate at 20°C for 10 min.
  d. Transfer the tube on a magnet and incubate at 20°C for 5 min.
  e. Withdraw the supernatant.
  f. Add 1 mL of 80% ethyl alcohol to the tube, without removing it from the magnet.
  g. Incubate for 30 s and repeat step 59e.-g.
  h. After two washes, remove the tube from the magnet.
  i. Briefly centrifuge the tube and place back on the magnet.
  j. Remove any remaining ethyl alcohol with a small pipette and let it dry for 15 min.
    **CRITICAL:** Remaining ethyl alcohol may negatively affect the final RPE product.
  k. Take the tube off the magnet and resuspend the beads with 40 μL of Elution Buffer.
  l. Incubate at 20°C for 2 min.
  m. Prepare a new 1.5 mL LoBind tube and label with “purified RPE product”.
  n. Place the tube with the beads on the magnet for 30 s.
  o. Transfer the clear solution into the new LoBind tube.
60. Perform the RPE PCR.
  a. Prepare the RPE PCR mix (Table 7) in pre-PCR workspace by combing all reagents into one 1.5 mL LoBind tube. Vortex-mix, briefly centrifuge and bring to post-PCR amplification workspace.

**Table 7:** RPE PCR mix

Component	Amount [μl] for 1 sample with no overage
PCR Master Mix	60.0
Universal Oligo	10.0
WTA Amplification Primer	10.0
  b. Add 40 μL of the “purified RPE product” (step 59) to the RPE PCR mix tube and pipet-mix 10 times.
  c. Pipet 60 μL RPE PCR reaction into each of two PCR tubes.
  d. Centrifuge the PCR strip and start the thermal cycler according to following program (Table 8).
    ***Note:*** The number of PCR cycles depend on the number of cells retrieved from the cartridge. We recommend 11 cycles for >20,000 cells, 12 cycles for >10,000 and 13 cycles for >5,000 cells.
    **Pause point:** Run PCR overnight at 4°C.

**Table 8:** PCR cycling conditions for RPE PCR

Step	Temperature	Time	Cycles
Hot Start	95°C	3 min	1
Denaturation	95°C	30 s	11–13x
Annealing	60°C	1 min	
Extension	72°C	1 min	
Final extension	72°C	2 min	1
Hold	4°C	N/A	N/A
61. (Optional) During RPE PCR perform the purification of the Sample Tag PCR1 product.
  a. Centrifuge the PCR tube strip with the Sample Tag PCR1 samples and combine in one 1.5 mL LoBind tube.
    ***Note:*** Work in post-amplification workspace. AMPure beads should be brought to 20°C and vortexed at full speed for 1 min before use.
  b. Add 360 μL of AMPure beads to the Sample Tag PCR1 product and pipet-mix 10 times.
  c. Incubate at 20°C for 5 min.
  d. Transfer the tube onto a magnet and incubate at 20°C for 5 min.
  e. Withdraw the supernatant.
  f. Add 500 μL of 80% ethyl alcohol to the tube, without removing it from the magnet.
  g. Incubate for 30 s and repeat step 61e.-g.
  h. Remove the tube from the magnet.
  i. Briefly centrifuge the tube and place back on the magnet.
  j. Remove any remaining ethyl alcohol with a small pipette and let it dry for 5 min.
  k. Take the tube off the magnet and resuspend the beads with 30 μL of Elution Buffer.
  l. Incubate at 20°C for 2 min.
  m. Prepare a new 1.5 mL LoBind tube and label with “purified Sample Tag PCR1 product”.
  n. Place the tube with the beads on the magnet for 30 s.
  o. Transfer the clear solution into the new LoBind tube.
    **Pause point:** store at −20°C for a maximum of 6 months or immediately perform Sample Tag PCR2.
62. After RPE PCR is complete, perform the purification of the RPE PCR product.
  a. Centrifuge the PCR tube strip with the RPE PCR samples and combine in one 1.5 mL LoBind tube.
    ***Note:*** AMPure beads should be brought to 20°C and vortexed at full speed for 1 min before use.
  b. Add 120 μL of AMPure beads to the RPE PCR product and pipet-mix 10 times.
  c. Incubate at 20°C for 5 min.
  d. Transfer the tube onto a magnet and incubate at 20°C for 3 min.
  e. Withdraw the supernatant.
  f. Add 500 μL of 80% ethyl alcohol to the tube, without removing it from the magnet.
  g. Incubate for 30 s and repeat step 62e.-g.
  h. Remove any remaining ethyl alcohol with a small pipette and let it dry for 5 min.
    **CRITICAL:** Remaining ethyl alcohol may negatively affect the final RPE product.
  i. Take the tube off the magnet and resuspend the beads with 40 μL of Elution Buffer.
  j. Incubate at 20°C for 2 min.
  k. Prepare a new 1.5 mL LoBind tube and label with “purified RPE PCR product”.
  l. Place the tube with the beads on the magnet for 30 s.
  m. Transfer the clear solution into the new LoBind tube.
    **Pause point:** Store at −20°C for a maximum of 6 months.
63. (Optional) Run Sample Tag PCR2 with “purified Sample Tag PCR1 product”.
  a. Prepare the Sample Tag PCR2 mix (Table 9) in pre-PCR workspace by combing all reagents in one 0.2 mL PCR tube. Vortex-mix, briefly centrifuge and bring to post-PCR amplification workspace.

**Table 9:** Sample Tag PCR2 mix

Component	Amount [μl] for 1 sample with no overage
PCR Master Mix	25.0
Nuclease-free water	15.0
Sample Tag PCR2 primer	3.0
Universal oligo	2.0
  b. Add 5 μL of the “purified Sample Tag PCR1 product” to 45 μL of Sample Tag PCR2 mix. Vortex-mix briefly.
  c. Centrifuge the PCR strip and start the thermal cycler according to following program (Table 10).

**Table 10:** PCR cycling conditions for Sample Tag PCR2

Step	Temperature	Time	Cycles
Hot Start	95°C	3 min	1
Denaturation	95°C	30 s	10x
Annealing	66°C	30 s	
Extension	72°C	1 min	
Final extension	72°C	5 min	1
Hold	4°C	N/A	N/A

**Pause point:** Run PCR overnight at 4°C.

**Figure 6:**
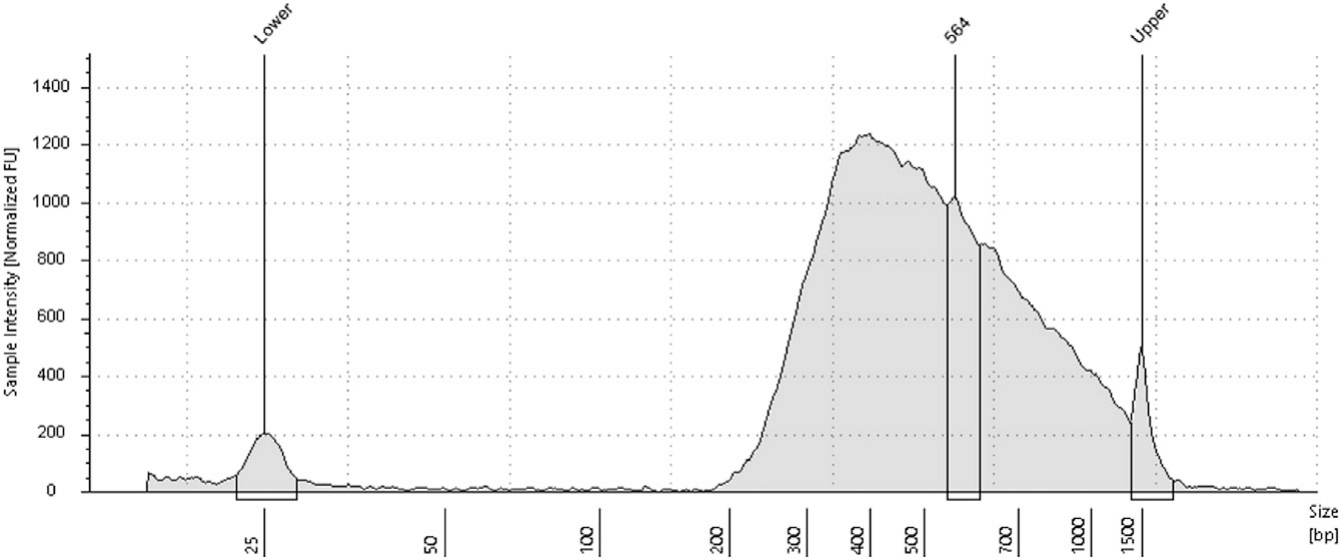
TapeStation High Sensitivity D1000 sample traces of purified RPE PCR product

64. (Optional) After Sample Tag PCR2 is complete, perform the purification of the PCR product.
  a. Centrifuge the PCR strip tube with the Sample Tag PCR2 sample.
    ***Note:*** Work in post-amplification workspace. AMPure beads should be brought to 20°C and vortexed at full speed for 1 min before use.
  b. Add 60 μL of AMPure beads to 50 μL of PCR product and pipet-mix 10 times.
  c. Incubate at 20°C for 5 min.
  d. Transfer the tube onto a magnet and incubate at 20°C for 3 min.
  e. Withdraw the supernatant.
  f. Add 200 μL of 80% ethyl alcohol to the tube, without removing it from the magnet.
  g. Incubate for 30 s and repeat step 64e.-g.
  h. Remove the tube from the magnet.
  i. Briefly centrifuge the tube and place back on the magnet.
  j. Remove any remaining ethyl alcohol with a small pipette and let it dry for 3 min.
    **CRITICAL:** Remaining ethyl alcohol may negatively affect the final Sample Tag PCR2 product.
  k. Take the tube off the magnet and resuspend the beads with 30 μL of Elution Buffer.
  l. Incubate at 20°C for 2 min.
  m. Prepare a new 1.5 mL LoBind tube and label with “purified Sample Tag PCR2 product”.
  n. Place the tube with the beads on the magnet for 30 s.
  o. Transfer the clear solution into the new LoBind tube.
    **Pause point:** Store at −20°C for max. 6 months.
65. Measure the quantity of the purified RPE PCR and Sample Tag PCR2 product using a Qubit fluorometer.
  a. Prepare the Qubit working solution according to the manufacturers protocol by diluting the dsDNA HS Reagent 1:200 with the dsDNA HS Buffer.
  b. Label the Qubit tubes on the lid.
  c. Add 190 μL of Qubit working solution to the standard tubes and 198 μL to the sample tube.
  d. Vortex and centrifuge the standards. Add 10 μL of each standard to the corresponding tubes.
  e. Centrifuge the purified PCR sample and add 2 μL to the corresponding tube.
  f. Vortex-mix all tubes and briefly centrifuge.
  g. Measure the concentration with the Qubit fluorometer.
  h. The concentration of the RPE PCR product should be between 0.5–10 ng/μL (see troubleshooting 2) and of the Sample Tag PCR2 product >0.1 ng/μL (see troubleshooting 3).
66. Measure the quality of the purified RPE PCR product with the TapeStation system.
  a. Add 2 μL of D1000 buffer to the tube strip.
  b. Add 2 μL of ladder or 2 μL of sample.
  c. Measure the quality with the 4200 TapeStation system.
  d. The peak should be within the range of 200–2,000 bp (see Figure 6).

### Preparation of the whole transcriptome mRNA library and Sample Tag library (optional)

**Timing: 4 h**

For sequencing with the Illumina platform, sequencing adapters and indices are added to the RPE PCR product and Sample Tag PCR2 product. Choose from 4 different reverse primers provided in the WTA Kit. The library preparation of the RPE PCR and Sample Tag PCR2 product from one experiment must be performed with the same reverse primer. Combine up to 4 experiments, using 4 different reverse primers for one Illumina sequencing run.

67. Ensure all the reagents below from the BD Rhapsody WTA Kit are fully thawed, vortexed and centrifuged. Place all tubes on ice.
  a. Nuclease-free water.
  b. PCR Master Mix.
  c. Library Forward Primer.
  d. Library Reverse Primer 1–4.
  e. Elution Buffer.
68. Dilute the purified RPE PCR product with Elution Buffer to 2 nM and perform WTA index PCR.
  a. Prepare the WTA index PCR mix (Table 11) in pre-PCR workspace by combing all reagents into a 0.2 mL PCR tube strip. Vortex-mix, briefly centrifuge and bring to post-amplification workspace.

**Table 11:** WTA index PCR mix

Component	Amount [μL] for 1 sample with no overage
PCR Master Mix	25.0
Nuclease-free water	5.0
Library Forward Primer	5.0
Library Reverse Primer 1–4	5.0
  b. Add 10 μL of the diluted “purified RPE PCR product” to the WTA index PCR mix tube and pipet-mix 10 times.
  c. Centrifuge the PCR strip and start the thermal cycler according to following program (Table 12).

**Table 12:** PCR cycling conditions for WTA index PCR

Step	Temperature	Time	Cycles
Hot Start	95°C	3 min	1
Denaturation	95°C	30 s	8–9x
Annealing	60°C	30 s	
Extension	72°C	30 s	
Final extension	72°C	1 min	1
Hold	4°C	N/A	N/A

***Note:*** The number of PCR cycles depends on the concentration of the diluted purified PCR product. We recommend 9 cycles if concentration is below 2 nM.

**Pause point:** Run PCR overnight at 4°C.

69. After WTA index PCR is complete, perform the dual-sided purification of the PCR product.
  a. Centrifuge the PCR tube strip with the WTA index PCR sample.
    ***Note:*** Work in post-amplification workspace. AMPure beads should be brought to 20°C and vortexed at full speed for 1 min before use.
  b. Add 60 μL of nuclease-free water to 50 μL of PCR product and pipet-mix 10 times.
  c. Prepare a new 0.2 μL PCR tube/strip and transfer 100 μL of the diluted WTA index PCR product.
  d. Add 60 μL of AMPure beads and pipet-mix 10 times.
  e. Incubate at 20°C for 5 min.
  f. Transfer the tube onto a magnet and incubate at 20°C for 2 min.
  g. During incubation, prepare a new 0.2 μL PCR tube and add 15 μL of AMPure beads.
  h. Transfer 160 μL of supernatant from step 69c.-d. to the new 0.2 μL PCR tube.
  i. Pipet-mix 10 times.
  j. Incubate at 20°C for 5 min.
  k. Transfer the tube on a magnet and incubate at 20°C for 1 min.
  l. Withdraw the supernatant.
  m. Add 200 μL of 80% ethyl alcohol to the tube, without removing it from the magnet.
  n. Incubate for 30 s and repeat step 69l.-n.
  o. Remove the tube from the magnet.
  p. Briefly centrifuge the tube and place back on the magnet.
  q. Remove any remaining ethyl alcohol with a small pipette and let it dry for max. 1 min.
    **CRITICAL:** Remaining ethyl alcohol may negatively affect the final WTA index PCR product.
  r. Take the tube off the magnet and resuspend the beads with 30 μL of Elution Buffer.
  s. Incubate at 20°C for 2 min.
  t. Prepare a new 1.5 mL LoBind tube and label with “purified WTA index PCR product”.
  u. Place the tube with the beads on the magnet for 30 s.
  v. Transfer the clear solution into the new LoBind tube.
    **Pause point:** Store at −20°C for max. 6 months.
70. (Optional) Dilute the Sample Tag PCR2 product with Elution Buffer to 1.1 ng/μL if required and perform Sample Tag index PCR.
  a. Prepare the Sample Tag index PCR mix (Table 13) in pre-PCR workspace by combing all reagents in a 0.2 mL PCR tube/strip. Vortex-mix, briefly centrifuge and bring to post-amplification workspace.

**Table 13:** Sample Tag index PCR mix

Component	Amount [μL] for 1 sample with no overage
PCR Master Mix	25.0
Nuclease-free water	18.0
Library Forward Primer	2.0
Library Reverse Primer 1–4	2.0
  b. Add 3 μL of the diluted “purified RPE product” to the Sample Tag index PCR mix tube to get a final volume of 50 μL and pipet-mix 10 times.
  c. Centrifuge the PCR strip and start the thermal cycler according to following program (Table 14).

**Table 14:** PCR cycling conditions for Sample Tag index PCR

Step	Temperature	Time	Cycles
Hot Start	95°C	3 min	1
Denaturation	95°C	30 s	6–8x
Annealing	60°C	30 s	
Extension	72°C	30 s	
Final extension	72°C	1 min	1
Hold	4°C	N/A	N/A

***Note:*** The number of PCR cycles depends on the concentration of the diluted purified PCR product. We recommend 6 cycles if concentration is below 1.1 ng/μL.

**Pause point:** Run PCR overnight at 4°C.

**Figure 7:**
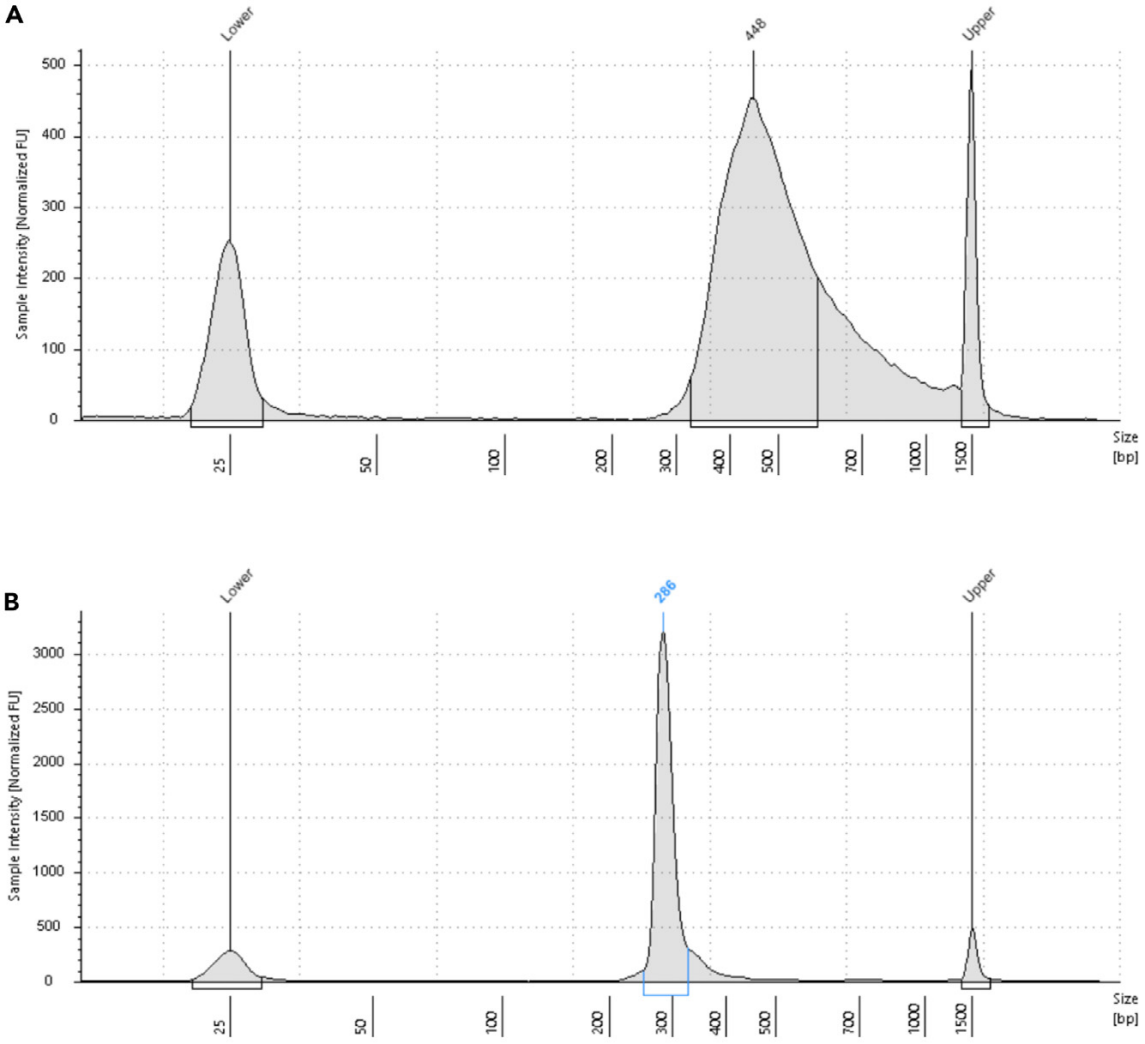
Qualitative analysis of WTA and Sample Tag index PCR products TapeStation High Sensitivity D1000 sample traces of WTA (A) and (optional) Sample Tag (B) library preparation.

71. (Optional) After Sample Tag index PCR is complete, perform the purification of the PCR product.
  a. Centrifuge the PCR tube strip with the Sample Tag index PCR sample.
    ***Note:*** Work in post-amplification workspace. AMPure beads should be brought to 20°C and vortexed at full speed for 1 min before use.
  b. Add 40 μL of AMPure beads to 50 μL of PCR product and pipet-mix 10 times.
  c. Incubate at 20°C for 5 min.
  d. Transfer the tube on a magnet and incubate at 20°C for 3 min.
  e. Withdraw the supernatant.
  f. Add 200 μL of 80% ethyl alcohol to the tube, without removing it from the magnet.
  g. Incubate for 30 s and repeat step 71e.-g.
  h. Remove the tube from the magnet.
  i. Briefly centrifuge the tube and place back on the magnet.
  j. Remove any remaining ethyl alcohol with a small pipette and let it dry for 3 min.
    **CRITICAL:** Remaining ethyl alcohol may negatively affect the final Sample Tag index PCR product.
  k. Take the tube off the magnet and resuspend the beads with 30 μL of Elution Buffer.
  l. Incubate at 20°C for 2 min.
  m. Prepare a new 1.5 mL LoBind tube and label with “purified Sample Tag index PCR product”.
  n. Place the tube with the beads on the magnet for 30 s.
  o. Transfer the clear solution into the new LoBind tube.
    **Pause point:** Store at −20°C for max. 6 months.
72. Measure the quantity of the purified WTA index and Sample Tag index PCR product with Qubit fluorometer as described in step 65. The expected concentration of the WTA index PCR product should be above 1 ng/μL and of the Sample Tag index PCR product above 1.5 ng/μL (see troubleshooting 4).
73. Measure the quality of the purified WTA index and Sample Tag index PCR product with TapeStation as described in step 66. The peak for the WTA index PCR product should be within the range of 250–1,000 bp. The Sample Tag index PCR product should be approximately 270 bp (see Figure 7 and troubleshooting 5).

### Sample pooling and sequencing requirements

**Timing: 1 h**

74. Prepare 1.5 mL LoBind tubes and dilute all samples to a final concentration of 400 nM.
75. Combine the diluted samples to a final pooled volume of 100 μL in a new 1.5 mL LoBind tube.
76. Sequence the libraries with paired-end Illumina sequencing. Use 50,000 reads per cell for WTA and 4,000 reads per cell for Sample Tag library sequencing.

### Data pre-processing with SevenBridges

**Timing: various**

77. Backup the sequencing data containing the fastq files (both R1 and R2 needed).
78. Set up the project for downstream analysis:
  a. Create a new account and project on the BD SevenBridges platform (http://www.sevenbridges.com/bdgenomics/).
  b. Upload your fastq files to the new project.
    ***Note:*** Depending on the size of your data, we recommend performing the upload via the command line (https://docs.sevenbridges.com/docs/upload-via-the-command-line).
  c. Add the BD Rhapsody Sequence Analysis Pipeline App (v1.2) to the project.
  d. Generate a new task by choosing “Run” in the “Apps” tab.
  e. Navigate to the new task and add the fastq reads (both R1 and R2) to the “Reads” tab as well as the desired reference genome to the “Reference Files Archived”.
  f. In the app settings, choose “Exact Cell Count” of 150,000 within the “Putative_Cell_Calling_Settings”.
    ***Optional:*** To enable Sample Tag calling, enable “Multiplex-Settings” and choose the “Single-Cell Multiplexing Kit – Human”, then add the sample tags and your experimental setup. To store sequence alignment data in a binary format, set the “Bam_Settings” to “True”.
    ***Note:*** The proposed exact cell count for filtering allows to retain cells with low mRNA content otherwise automatically removed within the pipeline.
79. Start the run, upon completion we recommend downloading all the resulting files including the pipeline html report and save them accordingly.

### Downstream data analysis

The following section describes a basic workflow using the previously downloaded .h5ad files from SevenBridges. This workflow focuses on the most important steps including data setup, demultiplexing, quality control, normalization, dimensionality reduction and clustering performed on single samples.

**Figure 8:**
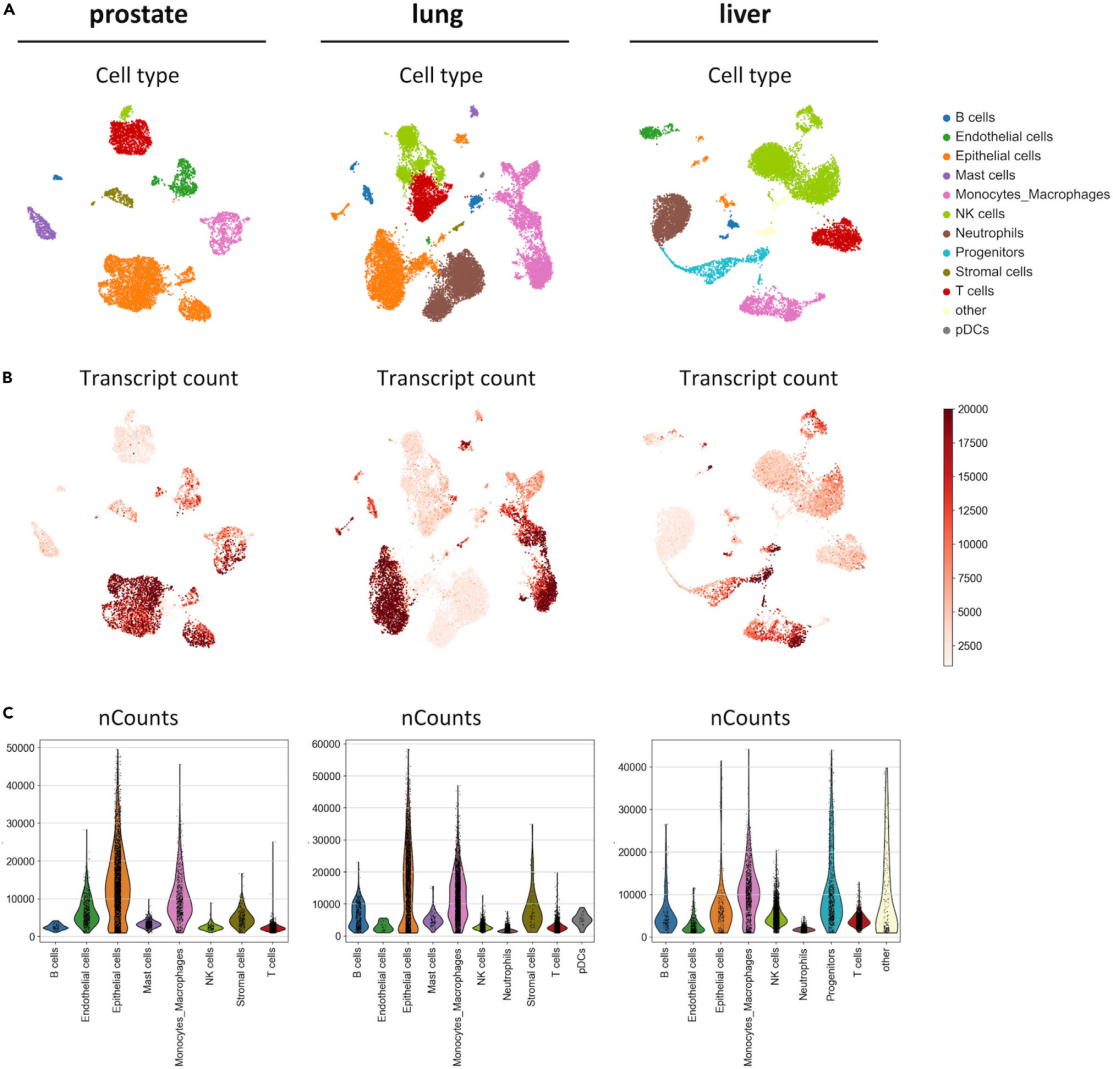
QC metrics of prostate (benign/tumor), lung (normal/tumor) and liver (normal) datasets generated with BD Rhapsody (A) Uniform manifold approximation and projection (UMAP) color-coded by cell type. (B) UMAP color-coded by total transcript counts. (C) nCounts quality metrics of filtered prostate, lung and liver cells.

***Note:*** For a detailed overview about the specific code used to generate the individual datasets for the studies, we refer to the original resources.^1^^,^^2^^,^^4^ To enable easy and fast analysis of scRNA-seq data, only main functions and dependencies included within the installation of the scanpy package (1.10.1) in python (> 3.6) are used. We highly recommend the scanpy documentation (https://scanpy.readthedocs.io/en/stable/) as well as the website from Hermos et al.^12^ which gets continuously updated (https://www.sc-best-practices.org). For illustration purposes, we provided the executed code as Jupyter notebook for each tissue type individually on Zenodo (see key resources table). Additionally, our GitHub page (https://github.com/UKIM-5/STAR_scRNA_seq) provides a more detailed version of the analysis including coarse cell annotation and improves visualizations, as an executable script to reproduce all the results visualized in this protocol. The results (Figures 8, S1, and S2) consist of individual samples taken from our published data including prostate, lung, and liver tissues. For data acquisition we used normal/benign and tumor tissue of prostate and lung, and only normal tissue of liver.

80. Analysis of raw .h5ad files from SevenBridges.
  a. Import needed modules and setup up the single-cell data object.
    i. Import modules.
      import scanpy as sc
      import pandas as pd
      import numpy as np
      import matplotlib.pyplot as plt
      import seaborn as sns
    ii. Read in the h5ad files, remove the placeholders with the file path to your stored data.
      file_path = "<userpath>lung_raw.h5ad"
      adata = sc.read_h5ad(file_path)
  b. Perform quality control by removing undetermined cells and filter cells based on thresholds.
    ***Optional:*** In case of multiplexed samples, setup up the data and only keep cells that were correctly assigned while removing the rest.
    adata.obs = adata.obs.iloc[:,[1,2]]
    adata = adata[(adata.obs["Sample_Name"] == "GG")|(adata.obs["Sample_Name"] == "TU")].copy()
    i. Identify mitochondrial genes and calculate quality control metrics.
      adata.var["mt"] = adata.var_names.str.startswith("MT-")
      sc.pp.calculate_qc_metrics(
       adata, qc_vars=["mt"], log1p=False, inplace=True, percent_top=None
      )
    ii. Plot the raw data using violin visualization to identify optimal thresholds.
      ***Optional:*** Remove the “#” in front of the group by command if samples were multiplexed.
      sc.pl.violin(
       adata,
       ["n_genes_by_counts", "total_counts", "pct_counts_mt"],
       #groupby="Sample_Name",
       size = 0.25,
       jitter=0.2,
       multi_panel=True,
      )
    iii. Perform filtering according to the selected filtering criteria and the samples individual needs, here we present the filters used for our liver sample.^4^
      sc.pp.filter_cells(adata, min_counts = 1000)
      sc.pp.filter_cells(adata, max_counts = 100000)
      sc.pp.filter_cells(adata, min_genes = 250)
      sc.pp.filter_cells(adata, max_genes = 8000)
      adata = adata[adata.obs["pct_counts_mt"] < 30].copy()
      ***Note:*** As described in a previous publication we choose a less stringent threshold of < 30% mitochondrial counts due to the increased presence of mitochondrial transcripts using the BD Rhapsody system.^1^
    iv. Visualize the filtered data again for comparison to the raw data.
      ***Optional:*** Remove the “#” in front of the group by command if samples were multiplexed.
      sc.pl.violin(
       adata,
       ["n_genes_by_counts", "total_counts", "pct_counts_mt"],
       #groupby=" Sample_Name ",
       size = 0.25,
       jitter=0.2,
       multi_panel=True,
      )
  c. Perform data scaling and transformation to remove technical biases and stabilize variance.
    i. Normalize the data and transform it using the log1p function but save the raw counts in a separate layer.
      adata.layers["counts"] = adata.X
      sc.pp.normalize_total(adata)
      sc.pp.log1p(adata)
    ii. Calculate highly variable genes based on Seurat_v3 flavor using raw counts stored in the count layer.
      sc.pp.highly_variable_genes(
       adata,
       layer = "counts",
       n_top_genes=4000,
       subset=False,
       flavor="seurat_v3",
      )
      ***Note:*** The ideally chosen number of selected variable genes is dependent on your sample; please refer to our published papers where you can find the chosen values for our datasets.^2^^,^^4^^,^^5^
  d. Reduce the data dimensionality and compute neighbors and embeddings for visualization as well as Leiden clustering.
    i. Calculate principal components for dimensionality reduction based on highly variable genes identified before.
      sc.tl.pca(adata, mask_var="highly_variable")
    ii. Compute the nearest neighbor distance matrix.
      sc.pp.neighbors(adata)
    iii. Embed the neighborhood graph using Uniform Manifold Approximation and Projection (UMAP).
      sc.tl.umap(adata)
    iv. Cluster the cells using the Leiden algorithm.
      sc.tl.leiden(adata, resolution=0.5)
    v. Visualize identified Leiden clusters and total counts using UMAP.
      sc.pl.umap(adata, color="leiden")
      sc.pl.umap(adata, color="total_counts")

## Expected outcomes

### Viable cell numbers after tissue dissociation

The dissociation protocols are optimized for each tissue. For healthy lung tissue, we expect to obtain approximately 7 × 10^6^ viable cells/g and for lung tumor tissue 12 × 10^6^ viable cells/g. Yields for viable liver cells will be approximately 3 × 10^6^ cells/g. As the prostate samples are obtained from punch biopsies, calculating the number of cells per gram is not feasible. However, we usually achieve approximately 1.3 × 10^5^ viable cells per healthy tissue biopsy and 1.7 × 10^5^ viable cells per tumor biopsy. Generally, cell numbers are higher for tumor tissues, due to leukocyte infiltration. However, viability is often low due to the common presence of necrosis.

### BD Rhapsody Cartridge loading

**Figure 9:**
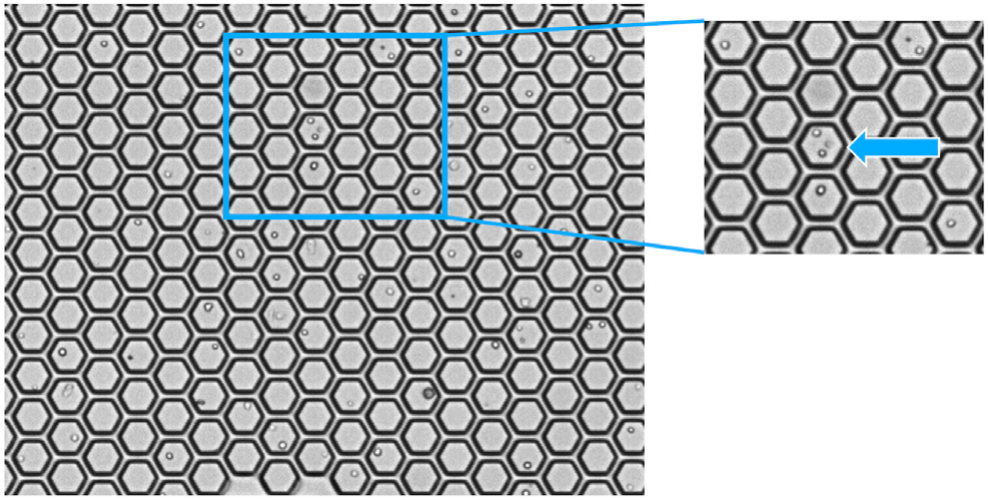
Image of the microwells in the BD Rhapsody Cartridge after cell load Multiplet indicated with an arrow.

Multiplets should be removed during bioinformatic analysis as they will interfere with interpretation of results. As shown in Table 15 the multiplet rate increases with the number of cells loaded. The multiplet rate estimated by BD is calculated by Poisson distribution using the number of cells loaded into the cartridge. Our experimental data indicates a slightly higher multiplet rate (Table 15). Figure 9 shows a representative cell load image of lung tissue with a number of viable cells captured in wells with a bead of 23,044 and a multiplet rate of 6.1% detected and calculated by the scanner. Sample Tags can help identify multiplets bioinformatically.

**Table 15:** Multiplet rate estimated vs. observed

Number of viable cells with bead	Multiplet rate estimated by BD	Multiplet rate observed^a^
10,000	2.4	3.3 ± 1.2 (*n* = 3)
15,000	3.5	4.8 ± 0.4 (*n* = 8)
20,000	4.7	6.3 ± 3.2 (*n* = 6)
25,000	N/A	6.5 ± 0.6 (*n* = 5)
30,000	N/A	7.0 ± 0.6 (*n* = 3)

a Mean and standard deviation of prostate, lung and liver samples.

### Single-cell RNA sequencing

The use of scRNA-seq from this protocol is anticipated to provide a comprehensive and detailed characterization of the immune cell populations present in the sample. This technique is highly effective in profiling immune cells, allowing for the identification and analysis of various immune cell types, states, and their interactions within the microenvironment. The detailed resolution offered by scRNA-seq will enable the detection of subtle differences in gene expression among immune cells, which can lead to the identification of novel cell subtypes and the elucidation of their roles in the biological context under investigation.

## Limitations

The BD Rhapsody platform excels in recovering cells with low mRNA content, such as leukocytes, while tending to underrepresent the epithelial compartment.^1^ This limitation must be considered when interpreting the results, particularly in studies where epithelial cell behavior and interactions are of potential interest. The microwell-based technology may lead to the loss of large cells (>40 μm), such as hepatocytes, due to a bead exclusion phenomenon based on well size.^4^ Further, not only the selection of the appropriate scRNA-seq technology is crucial, but tissue dissociation also significantly impacts outcome. Selecting the appropriate protocol for tissue dissociation is critical, as it impacts the cellular composition of the single-cell suspension and must be optimized for the specific tissue to be analyzed. The sample-multiplexing capability in the BD Rhapsody workflow by Sample Tags reduces technical bias caused by batch effects and substantially lowers sequencing library preparation costs. However, we could recently demonstrate that the Sample Tag staining procedure markedly impairs RNA quality in freshly isolated single-cell suspensions derived from complex tissues, potentially affecting sequencing library complexity and the overall outcome of the scRNA-seq experiment.^1^ Taken together, these critical decisions including which scRNA-seq technology, tissue dissociation as well as sample-multiplexing should be thoroughly considered, to finally select the most appropriate sample preparation protocol and technology platform for the specific experimental question to be answered.

## Troubleshooting

### Problem 1

**Figure 10:**
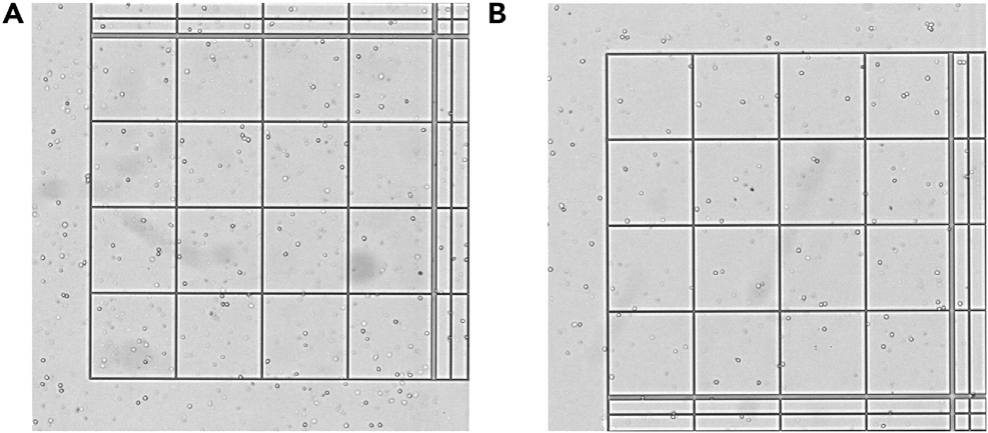
Results of erythrocyte lysis Cell count image before (A) and after (B) additional erythrocyte lysis.

High number of erythrocytes in cell count step after tissue dissociation (see Figure 10). Step 10 “cell counting”.

### Potential solution

Repeat erythrocytes lysis with BD Pharm Lyse lysing buffer. Be aware that each erythrocyte lysis is accompanied with a general loss of 5%–10% viability. Do not repeat in samples with low cell viability.

### Problem 2

The Random Priming and Extension PCR product is lower than expected. Step 65 “step-by-step method details”.

### Potential solution

Repeat the on bead random priming and extension (and Sample Tag denaturation) step.

### Problem 3

The purified Sample Tag PCR 2 product is lower than expected. Step 65 “step-by-step method details”.

### Potential solution

Repeat the Sample Tag PCR 2 and increase the PCR cycles by 1 or 2 cycles. If concentration of Sample Tag PCR 2 does not increase, repeat Sample Tag PCR1 with an additional PCR cycle and continue with Sample Tag PCR 2. If the concentration still remains low, repeat the Sample Tag denaturation from the bead. Repeat Sample Tag PCR 1 and PCR2.

### Problem 4

The purified index PCR product concentration is lower than expected. Step 72 “step-by-step method details”.

### Potential solution

Repeat the index PCR amplification and increase the PCR cycles. Do not extend the final cycle number to more than 2 extra cycles. Additional PCR amplification cycles might add an PCR bias to the final product.

### Problem 5

**Figure 11:**
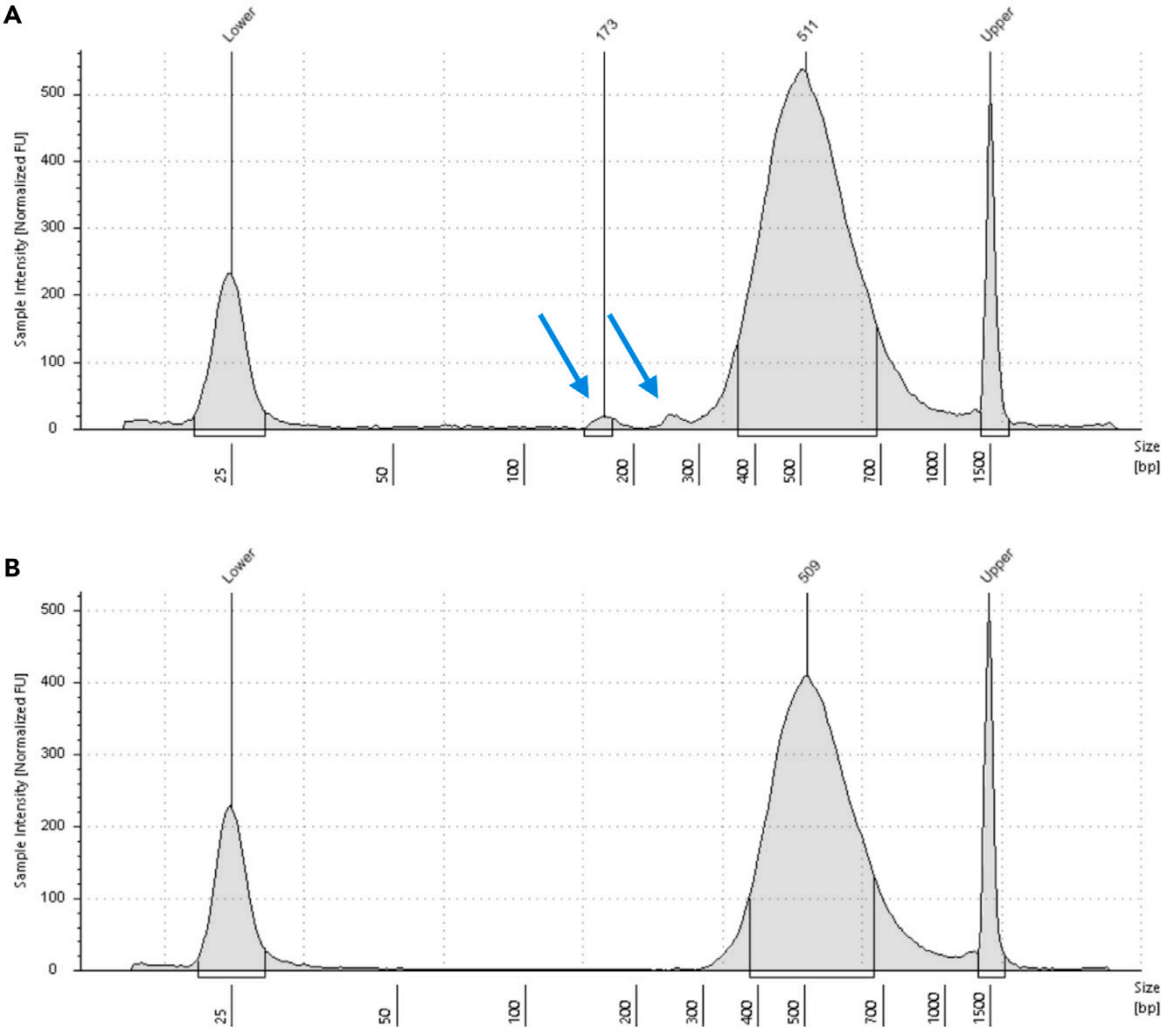
Qualitative analysis of PCR product purification TapeStation profiles of purified WTA index PCR product before (A) and after (B) additional purification.

The TapeStation cDNA quality measurement of the purified PCR product shows additional peaks to the expected main peak (see Figure 11). Step 73 “step-by-step method details”.

### Potential solution

If DNA yield is high, repeat the corresponding purification step. An additional purification leads to a loss of DNA. Repeat the PCR amplification and the purification if DNA yield is low. Adhere to the specified incubation times during purification. Try to avoid any residual volume during supernatant removal.

## Resource availability

### Lead contact

Stefan Salcher, stefan.salcher@i-med.ac.at.

### Technical contact

Alexandra Scheiber, alexandra.scheiber@i-med.ac.at.

### Materials availability

This study did not generate new unique reagents.

### Data and code availability

The published article includes all code generated during this study. Optional code for more extensive data processing and visualization are available at GitHub: https://github.com/UKIM-5/STAR_scRNA_seq. All data files used for generating results have been deposited at Zenodo: https://doi.org/10.5281/zenodo.12164999.

## Acknowledgments

This work was supported by the Austrian Science Fund FWF (grant no. TAI-697) (D.W.), the “In Memoriam Dr. Gabriel Salzner Stiftung” (D.W. and I.H.), the FFG grant of the Austrian Research Promotion Agency (grant no. 858057, HD FACS) (S. Sopper), the TWF grant (grant no F.16733/5–2019) (I.H.), the Tiroler Wissenschaftsfond (T.H.), and the Jubiläumsfonds — Österreichische Nationalbank (R.O.). We also want to thank the pathologists of the INNPATH GmbH, Institute of Pathology in Innsbruck.

## Author contributions

Conceptualization, S. Salcher, A. Pircher, and A.S.; sample preparation, A.S., M.T., and A. Pittl; scRNA sequencing, A.S., M.T., and S. Salcher; data analysis, A.S., M.T., S. Salcher, Z.T., and A. Pittl; human samples, F.A.; original draft writing, A.S. and M.T.; review and final editing, all authors; funding acquisition, I.H., T.H., R.O., S. Sopper, and D.W.

## Declaration of interests

The authors declare no competing interests.
