# Supplementary material for: Profiling low-mRNA content cells in complex human tissues using BD Rhapsody single-cell analysis
Source: STAR Protoc. 2024 Dec 9;5(4):103475. doi: 10.1016/j.xpro.2024.103475 (PMC11683236; doi:10.1016/j.xpro.2024.103475)
Supplement: Document S1. Figures S1 and S2 [file mmc1.pdf]

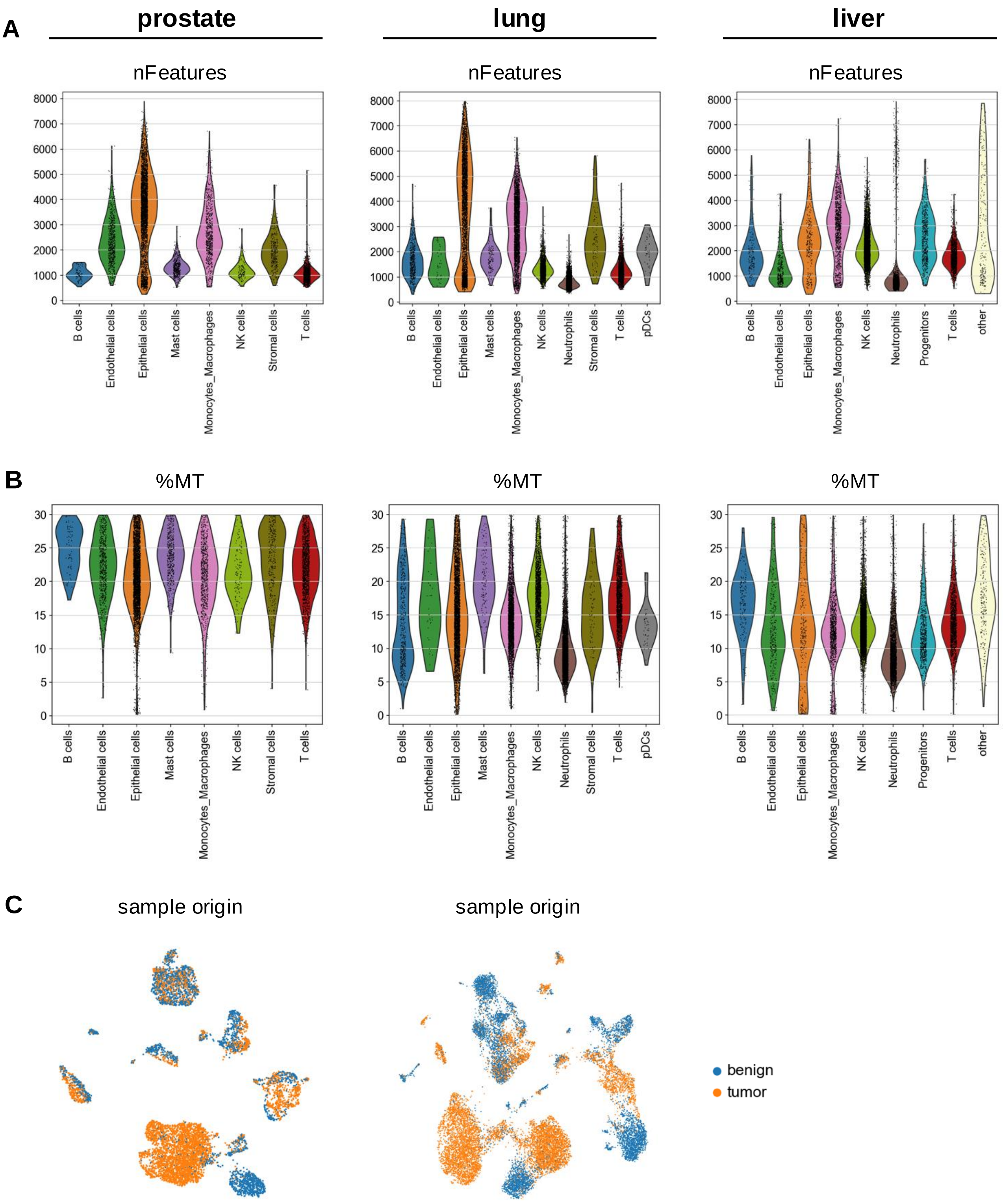

Figure S1: Related to step 80. Additional QC metrics of prostate (benign/tumor), lung (normal/tumor), and liver (normal) datasets generated with BD Rhapsody.

(A) nFeatures and (B) percentage of mitochondrial transcripts (%MT) quality metrics of filtered prostate, lung, and liver cells.

(C) UMAP plots of prostate and lung tissue cells, color-coded by origin: normal/benign (blue) and tumor (orange) tissue.

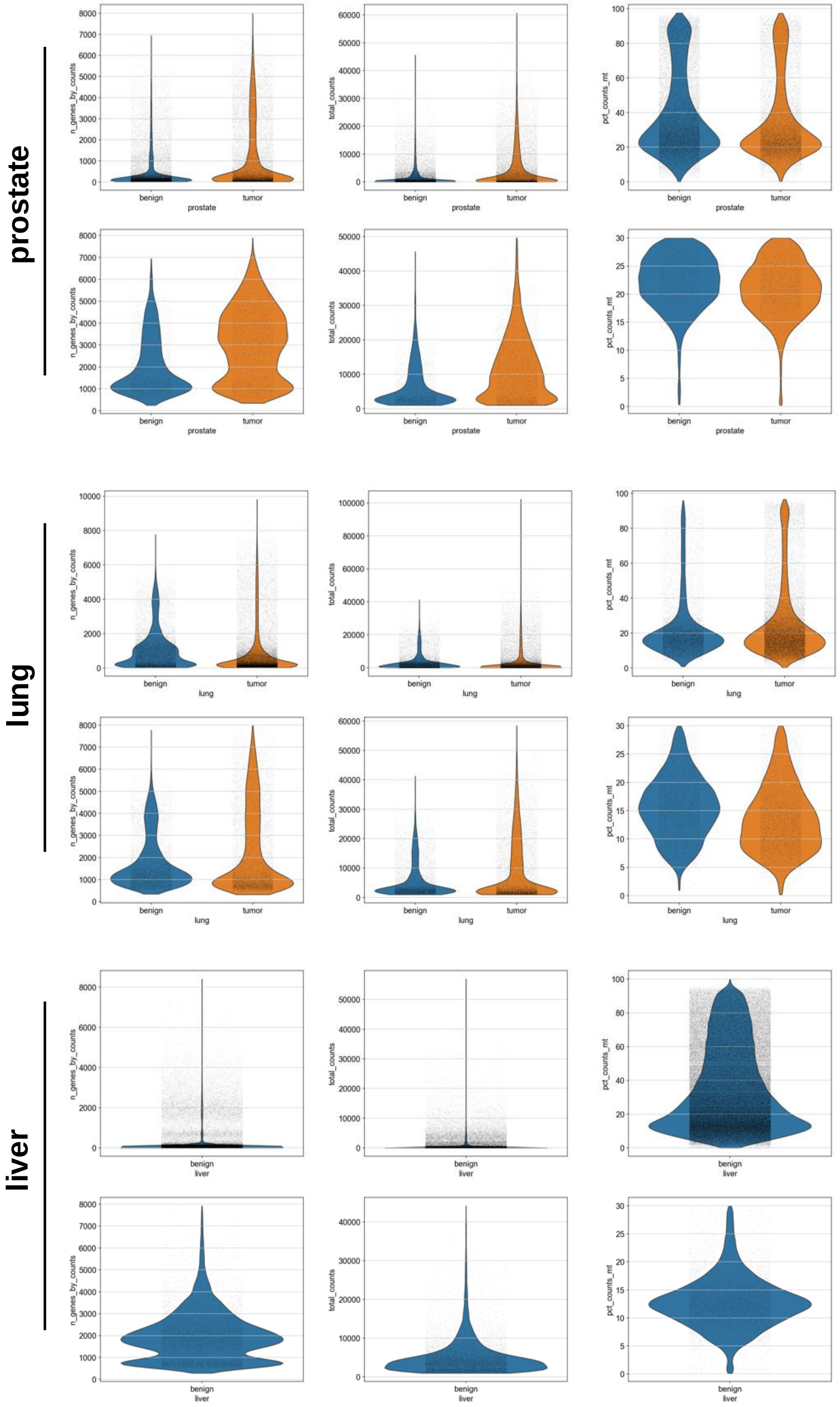

Figure S2: Related to step 80. Violin plots of prostate (benign/tumor), lung (normal/tumor), and liver (normal) datasets generated with BD Rhapsody before and after filtering.

The upper plots for each tissue show gene counts, total counts, and percentage of mitochondrial counts before filtering; the lower plots show these metrics after filtering. Normal/benign (blue) and tumor (orange) tissue.
